# Community indicators for mental health in Europe: a scoping review

**DOI:** 10.3389/fpubh.2023.1188494

**Published:** 2023-07-19

**Authors:** Petra Schoenweger, Michaela Kirschneck, Katharina Biersack, Anna-Francesca Di Meo, Philipp Reindl-Spanner, Barbara Prommegger, Claudia Ditzen-Janotta, Peter Henningsen, Helmut Krcmar, Jochen Gensichen, Caroline Jung-Sievers, for the POKAL-Group, Jochen Vukas

**Affiliations:** ^1^Institute of Medical Data Processing, Biometrics and Epidemiology (IBE), Faculty of Medicine, LMU Munich, Munich, Germany; ^2^Pettenkofer School of Public Health, Munich, Germany; ^3^Department of Psychosomatic Medicine and Psychotherapy, University Hospital, Technical University of Munich, Munich, Germany; ^4^TUM School of Computation, Information and Technology, Technical University of Munich, Munich, Germany; ^5^Institute of General Practice and Family Medicine, University Hospital of Ludwig-Maximilians-University Munich, Munich, Germany

**Keywords:** public mental health, community indicators, neighborhood, mental health promotion and prevention, scoping review

## Abstract

**Background:**

Community indicators may predict and influence individuals` mental health, and support or impede mental health management. However, there is no consensus on which indicators should be included in predictions, prognostic algorithms, or management strategies for community-based mental health promotion and prevention approaches. Therefore, this scoping review provides an overview of relevant community-level indicators for mental health in the general as well as risk populations in a European context.

**Methods:**

We conducted a scoping review in the following electronic databases: PubMed, Embase, and PsycInfo. Eligible studies focused on context factors such as either the physical or social environment, reporting at least one mental health outcome and referring to a European population. Publications between 2012 and March 8, 2022 are considered.

**Results:**

In total, the search yielded 12,200 identified records. After the removal of duplicates, 10,059 records were screened against the eligibility criteria. In total, 169 studies were included in the final analysis. Out of these included studies, 6% focused on pan-European datasets and 94% on a specific European country. Populations were either general or high-risk populations (56 vs. 44%, respectively) with depressive disorder as the main reported outcome (49%), followed by general mental health (33%) and anxiety (23%). Study designs were cross-sectional studies (59%), longitudinal (27%), and others (14%). The final set of indicators consisted of 53 indicators, which were grouped conceptually into 13 superordinate categories of community indicators. These were divided into the domains of the physical and social environment. The most commonly measured and reported categories of community indicators associated with mental health outcomes were social networks (*n* = 87), attitudinal factors toward vulnerable groups (*n* = 76), and the characteristics of the built environment (*n* = 56).

**Conclusion:**

This review provides an evidence base of existing and novel community-level indicators that are associated with mental health. Community factors related to the physical and social environment should be routinely recorded and considered as influencing factors or potentially underestimated confounders. The relevance should be analyzed and included in clinical outcomes, data, monitoring and surveillance as they may reveal new trends and targets for public mental health interventions.

## 1. Introduction

Over the last decades, mental health impairments have imposed a high burden on society and health care systems across European countries and worldwide in terms of premature mortality, increased morbidity, and decreased quality of life (1). Before the COVID-19 pandemic, an average of one in nine adults had symptoms of psychological distress and one in six adults was diagnosed with a mental health condition, with varying prevalence across Europe (2). The Global Burden of Disease Study 2015 (3) showed that among all mental health conditions, depression and anxiety disorders are most prevalent. It further showed that mental disorders were among the most important contributors to years of life with disability (DALYs) in 2015.

To reduce the high prevalence and high burden imposed by mental health disorders, large scale and effective mental health promotion and prevention strategies are necessary. These strategies should aim at avoiding mental ill-health in the first place and at reducing the impacts of mental disorders on people’s lives. The importance of mental health promotion and prevention was underlined by an analysis of the Australian mental health strategy between 1992 and 2011: while the expenditure on mental health services, workforce, and treatments increased dramatically, the prevalence of depression and anxiety disorders remained high and even showed some increase at the same time. A possible explanation, among others, was that comprehensive and on-going mental health promotion and prevention interventions were missing in the strategy (4). This explanation is supported by findings such as that mental health promotion and prevention programs showing to strengthen protective factors for mental health but often target specific populations rather than the general population (5).

Communities (or herein also referred to as neighborhoods) qualify as ideal settings for mental health promotion and prevention strategies. Moreover, the WHO Comprehensive Plan of Action 2013–2020 demands the re-shaping of community and natural environments as central pillars for improving the mental health of the population. The WHO calls it “misplaced priorities” as 2/3 of the money spent in mental health is currently allocated to psychiatric hospitals instead of supporting other key factors such as communities. The advantages of community-based mental health care, however, are more fundamental, including increased accessibility, reduced stigmatization, better protected human rights, and improved outcomes (6). In communities, health messages are received in the immediate environment and are therefore powerful in influencing the individual’s health behavior and behavior change (7). As people are inextricably linked to their environment, and health promotion and prevention moved already beyond the clinical service provision, the community should be involved as an effective partner (8). As pointed out by the WHO, changes in the transforming field of mental health care are not happening fast enough, and community-level mental health services should be strengthened to serve people best with low threshold access (6). Moreover, community-level factors should be acknowledged as potential targets of intervention and support of treatment and management concepts of mental health disorders (9).

In the management of chronic diseases, the integration of the community and its resources is a central pillar as conceptualized, for instance, within the Chronic Care Model (10). To meet the requirements and incorporate the principles in the field of health promotion and prevention, Barr et al. (11) developed the Expanded Chronic Care Model (ECCM), which focuses on the social determinants of health and recognizes the importance of socio-economic factors as a key determinant to long-term success in the prevention and treatment of mental diseases (8). The ECCM provides a strategy to support high-quality healthcare services with policies and programs in the communities to reduce the impact on those who are ill, but also to help people to stay healthy (11). For example, ECCM-based chronic disease programs for hypertension and diabetes in Canada integrated community resources by strengthening community action (e.g., developing a community network on the condition, bringing services to marginalized groups, shifting decision-making to adapt to community needs and capacities, partnering with local businesses, and involving the non-profit sector to develop a strategic vision) (12, 13). In the mental health field, linking patients to community-based assets, such as community music, community exercise, museums/arts, libraries, and gardening is increasingly used and could be a promising intervention to reduce depression and anxiety (14). For example, horticultural therapy included tours of a botanical garden, learning about the species and how to cultivate, grow, and harvest them, combined with activities such as planting, arranging flowers, etc. (15, 16).Knowing the pertinent contributing factors and indicators for community mental health is essential for the development of successful mental health promotion and prevention interventions. However, there is currently no consensus or solid evidence base on which community indicators are the most relevant and should be included in monitoring, surveillance (predictions, prognostic algorithms), or management data collections. Factors of the physical environment include the built environment, which is defined most generally as “man-made infrastructure” such as buildings and streets (17), public transportation infrastructure, and recreational sites and structures among others (18). The social environment considers the sociodemographic composition of an area, as well as “the relationships, groups and social processes that exist between individuals” including social capital, social norms, safety, and poverty (19). Generaal et al. (20) showed that these socioeconomic, physical, and social community or neighborhood characteristics are associated with the prevalence and severity of depressive disorders. Particularly, social cohesion, a defining feature of a contextual unit with its core dimensions of social relation, identification with the social entity, and orientation toward a common good (21, 22) mediates the effects of environmental and built neighborhood characteristics on physical and mental health with a proportion between 10 and 23% (23). In addition, social capital and the strongly correlating concept of social justice, a central cornerstone of community psychology, have a strong impact on wellbeing and shows a high effect on life satisfaction on a national level (24). The citizen’s wellbeing is strongly related to their mattering and social environment in the community (25).

While much literature omits the definition of an indicator but focuses straight on its qualities, Kaye-Blake et al. (26) define an indicator as a “relevant variable, measurable over time and/or space that provides information on a larger phenomenon of interest and allows comparisons to be made.” Hence, community indicators quantify different aspects of community characteristics over time. The implementation and integration of health promotion principles into the prevention and management of mental health problems depends upon a good understanding of the specific social and physical indicators regarding the individual and its (health) context (11). Therefore, the aim of this scoping review is to:

1. Systematically identify relevant indicators that predict, explain, or monitor neighborhood/community characteristics that are relevant for mental health.
2. Assign the extracted indicators to core categories and possibly even deductively identify new core areas in field of mental health associated community characteristics.
3. Identify gaps in the field and new targets of interventions in community based (public) mental health.

## 2. Materials and methods

### 2.1. Study design

The methodological steps are structured according to the suggestions of the “Methodology for Joanna Briggs Institute Scoping Reviews” (27) and its update (28). This is on the basis of the framework for scoping reviews by Arksey and O` Malley (29) and consists of the following steps: (1) Identify the research question, (2) Identify relevant studies, (3) Select studies, (4) Chart the data, and (5) Collate, summarize, and report the results.

Additionally, we employed the Preferred Reporting Items for Systematic reviews and Meta-Analyses (PRISMA) extension for Scoping Reviews Checklist (PRISMA-ScR Checklist) (30) as a guideline to develop this scoping review and the corresponding pre-registered study protocol. The study protocol was published in advance and can be viewed in the Open Science Framework at this link: https://osf.io/r6ngq.

### 2.2. Search strategy

We used the Population-Concept-Context (PCC) framework to build our search strategy and to guide the literature search considering: P (Population): general or high-risk community members/people living in a defined geographical area; C (Concept): community-level indicators/neighborhood characteristics, environmental determinants. C (Context): Mental health promotion and prevention in Europe (see Supplementary Table 1). For this paper, we focused on the term “community” in the sense of people living in a defined residential area, neighborhood, or town. For the sake of this work, we defined an indicator according to Kaye-Blake et al. (26) as a “relevant variable, measurable over time and/or space that provides information on a larger phenomenon of interest and allows comparisons to be made” and that is associated with the community’s mental health. But it was not the aim of this work to determine whether it fulfilled the criteria of a good indicator (quality appraisal) according to the current literature. To address heterogeneity regarding the healthcare, environmental and political systems, we only included studies that are based on European datasets. We conducted the search in three electronic databases (PubMed, Embase, and PsycInfo) on the March 8, 2022. The inclusion and exclusion criteria are described below. We chose the precise search terms according to the database used but all included title/abstract phrases such as “community,” “neighborhood,” “residential,” “environmental,” “indicators,” “characteristics,” “determinants,” “factors,” “mental health,” “depression,” “depressive disorders,” “anxiety,” and “mental disorders.” We give an example of the chosen keywords for our Medline search in the Supplementary Table 2. For the sake of completeness, we included a great variety of search terms to fully represent the topic. To account for the different types of evidence, we included quantitative and qualitative research. We excluded study protocols, case studies, conference abstracts, or gray literature.

### 2.3. Study selection with inclusion and exclusion criteria

To be included, the articles had to be (a) in English or German, (b) located in community or neighborhood, (c) focus on a European population, (d) focus on mental health outcomes, and (e) published between January 2012 and March 8, 2022 to retrieve recent publications and to depict current circumstances. Studies were excluded if they (a) were conducted in an institutional setting such as health care setting, school, workplace, or prison, (b) were occupation-related, (c) were COVID-19 related, (d) included the digital environment, such as social media platforms, online peer groups, and digital support systems as it cannot be geographically localized, (e) included medical interventions or health promoting activities (e.g., football training, mindfulness interventions) as these do not qualify as community indicator, and (f) included non-European data.

### 2.4. Data extraction process

**Figure 1:**
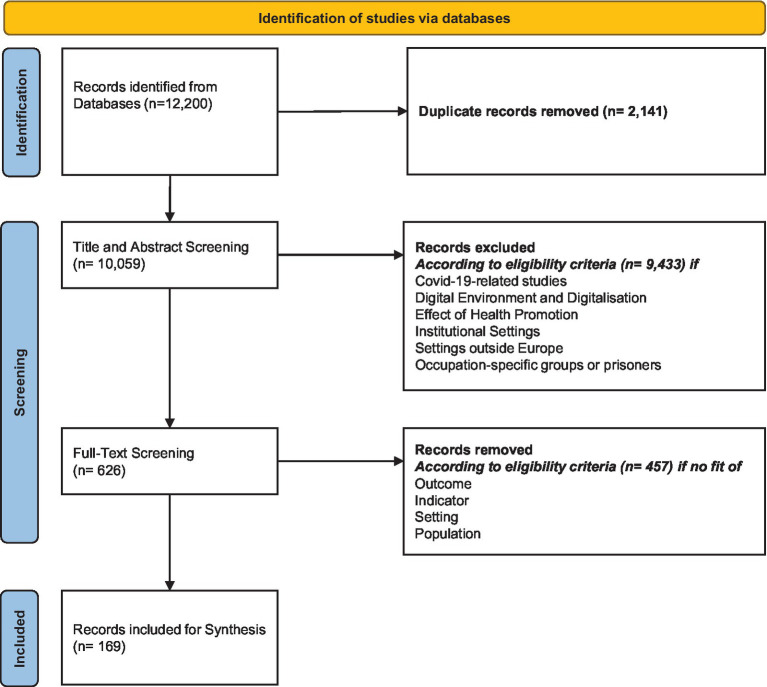
Flow chart.

All records retrieved in the electronic databases were merged using the Rayyan software. We deleted duplicates and the remaining records were first title and abstract screened, and then full-text screened against the inclusion and exclusion criteria. This process is shown in the PRISMA Flow Chart (Figure 1).

All records were distributed among the five reviewers (PS, KB, ADM, MK, and PRS) for screening using the Rayyan software. Twenty percent of the records from the title and abstract screen were double screened by two reviewers (PS, KB). Disagreements were resolved through discussion. The same five reviewers simultaneously conducted the full-text screen and data extraction.

For the data extraction, we developed a characterization matrix in an Excel spreadsheet, which comprised: first author, publication year, indicator(s), region(s), population, outcome measure(s), and study design (categorized as cross-sectional studies, longitudinal studies, and other study designs). In the data synthesis, we assigned the extracted indicators to either the physical or the social environment. If composite indicators were used in the studies, we extracted every single sub-indicator and regarded it as standalone indicator. We clustered conceptually similar indicators into superordinate indicator categories.

## 3. Results

### 3.1. Literature search

A total of 12.200 records were identified from the electronic databases. After removal of duplicates and records that did not meet the inclusion criteria, 169 studies were included in the final synthesis. Figure 1 gives an overview of the reasons for exclusion for each step of the screening process. The baseline characteristics of all studies are shown in the Table 1.

**Table 1:** Overview of included studies.

Author/Year	Study type	Region	Population		Community indicator categories
			High risk	Age group	
(31)	Systematic review	Norway	Yes	Adults	Attitudinal factors toward vulnerable groups
					Built environment
					Social networks
(32)	Cross-sectional study	Netherlands	Yes	Adolescents	Attitudinal factors toward vulnerable groups
					Built environment
					Deprivation
					Social networks
(33)	Cross-sectional study	Netherlands	Yes	Adolescents	Attitudinal factors toward vulnerable groups
					Built environment
					Deprivation
(34)	Review	Sweden	No	Adults	Social networks
(35)	Cross-sectional study	Germany	Yes	Adults	Attitudinal factors toward vulnerable groups
(36)	Cross-sectional study	Sweden	No	Adolescents	Social networks
(37)	Longitudinal study	United Kingdom	No	Adults	Built environment
(38)	Qualitative study	United Kingdom	No	Adults	Security
(39)	Longitudinal study	Croatia	No	Adults	Climate change
					Social networks
(40)	Case–Control	Croatia	Yes	Adults	Social networks
(41)	Cross-sectional study	United Kingdom	Yes	Adults	Deprivation
					Social networks
(42)	Longitudinal study	Europe	No	Adults	Access to services
					Pollution
(43)	Qualitative study	United Kingdom	No	Adults	Built environment
(44)	Cross-sectional study	Spain	No	Children	Deprivation
(45)	Cross-sectional study	Portugal	No	Adults	Access to services
					Presence of resources
(46)	Systematic review	Denmark Finland Norway Sweden	Yes	Adults	Attitudinal factors toward vulnerable groups
					Social networks
(47)	Cross-sectional study	Ukraine	No	Adults	Security
(48)	Cross-sectional study	Finland	Yes	Adults	Attitudinal factors toward vulnerable groups
(49)	Cross-sectional study	United Kingdom	Yes	Adolescents	Security
(50)	Cross-sectional study	Spain Europe	No	Adults	Macroeconomic environment
(51)	Ecological study	Germany	Yes	Adults	Built environment
					Population structure
(52)	Cross-sectional study	Netherlands	No	Adults	Social networks
(53)	Longitudinal study	United Kingdom	No	Adults	Deprivation
(54)	Cross-sectional study	United Kingdom	Yes	Adolescents	Attitudinal factors toward vulnerable groups
(55)	Meta-analysis	Europe	Yes	Adults	Attitudinal factors toward vulnerable groups
(56)	Cross-sectional study	Netherlands	No	Adults	Built environment
					Social networks
(57)	Cross-sectional study	Europe	Yes	Adolescents	Social networks
(58)	Longitudinal study	Norway	No	Adolescents	Social networks
(59)	Cross-sectional study	Belgium	Yes	Adults	Attitudinal factors toward vulnerable groups
					Social networks
(60)	Cross-sectional study	Finland Poland Spain	No	Adults	Built environment
					Mobility
					Social networks
(61)	Ecological study	Italy	Yes	Adults	Access to services
					Presence of resources
(62)	Cross-sectional study	Cyprus	No	Adults	Macroeconomic environment
					Social networks
(63)	Cross-sectional study	Greece	No	Adults	Macroeconomic environment
					Social networks
(64)	Mixed methods	United Kingdom	No	Adults	Social networks
(65)	Longitudinal study	Denmark	No	Adults	Built environment
(66)	Longitudinal study	Denmark	No	Children	Built environment
(67)	Longitudinal study	Spain Netherlands	No	Children	Pollution
(68)	Cross-sectional study	Netherlands	No	Adults	Access to service
					Built environment
					Mobility
					Presence of resources
					Population structures
					Social networks
(69)	Longitudinal study	United Kingdom	No	Adults	Deprivation
(70)	Longitudinal study	United Kingdom	No	Adults	Social networks
(71)	Cross-sectional study	Finland Sweden	No	Adults	Social networks
(72)	Longitudinal study	United Kingdom	Yes	Adults	Access to services
					Presence of resources
(73)	Cross-sectional study	Portugal	Yes	Adolescents	Attitudinal factors toward vulnerable groups
(74)	Cross-sectional study	Norway	Yes	Adults	Attitudinal factors toward vulnerable groups
(75)	Longitudinal study	Germany	No	Adults	Pollution
(20)	Cross-sectional study	Netherlands	No	Adults	Built environment
					Deprivation
					Pollution
					Security
					Social networks
(76)	Cross-sectional study	Netherlands	No	Adults	Built environment
					Deprivation
					Pollution
					Security
					Social networks
(77)	Cross-sectional study	United Kingdom	Yes	Adults	Attitudinal factors toward vulnerable groups
					Security
					Social networks
(78)	Longitudinal study	Finland	No	Adults	Built environment
(79)	Longitudinal study	Netherlands	No	Adolescents	Built environment
(80)	Cross-sectional study	United Kingdom	No	Adults	Attitudinal factors toward vulnerable groups
(81)	Longitudinal study	Sweden	No	Adults	Deprivation
					Social networks
(82)	Cross-sectional study	Norway	Yes	Adults	Attitudinal factors toward vulnerable groups
(83)	Cross-sectional study	United Kingdom	Yes	Adults	Attitudinal factors toward vulnerable groups
(84)	Cross-sectional study	Netherlands	Yes	Adults	Attitudinal factors toward vulnerable groups
					Social networks
(85)	Cross-sectional study	Austria	Yes	Adults	Built environment
(86)	Systematic review	Germany	Yes	Adults	Attitudinal factors toward vulnerable groups
(87)	Cross-sectional study	Netherlands	Yes	Adults	Attitudinal factors toward vulnerable groups
(88)	Cross-sectional study	Italy	No	Adults	Social networks
(89)	Longitudinal study	United Kingdom	Yes	Adults	Attitudinal factors toward vulnerable groups
(90)	Cross-sectional study	United Kingdom	No	Adults	Social networks
					Security
(91)	Cross-sectional study	United Kingdom	No	Adolescents	Deprivation
					Population structure
					Security
(92)	Longitudinal study	Sweden	No	Adults	Security
(93)	Cross-sectional study	Sweden	No	Adolescents	Social networks
(94)	Other	Germany	No	Adults	Built environment
(95)	Longitudinal study	United Kingdom	No	Adults	Mobility
(96)	Cross-sectional study	France	Yes	Adults	Security
(97)	Longitudinal study	Greece	Yes	Adults	Social networks
(98)	Cross-sectional study	Netherlands	No	Adults	Security
(99)	Longitudinal study	United Kingdom	Yes	Adults	Deprivation
(100)	Qualitative study	Ireland	No	Adults	Attitudinal factors toward vulnerable groups
(101)	Longitudinal study	Sweden	No	Adults	Social networks
(102)	Cross-sectional study	Europe	No	Adults	Attitudinal factors toward vulnerable groups
					Social networks
(103)	Longitudinal study	United Kingdom	No	Children	Pollution
(104)	Cross-sectional study	Sweden	Yes	Adults	Built environment
					Deprivation
					Social networks
(105)	Cross-sectional study	Romania Bulgaria	Yes	Adults	Attitudinal factors toward vulnerable groups
(106)	Cross-sectional study	Europe	Yes	Adults	Attitudinal factors toward vulnerable groups
					Policy
(107)	Cross-sectional study	Sweden	No	Adults	Social networks
					Deprivation
(108)	Longitudinal study	United Kingdom	No	Adults	Social networks
					Macroeconomic environment
(109)	Cross-sectional study	Spain	No	Adults	Built environment
					Population structure
(110)	Cross-sectional study	Spain	No	Adults	Attitudinal factors toward vulnerable groups
(111)	Cross-sectional study	Spain	Yes	Adults	Attitudinal factors toward vulnerable groups
(112)	Longitudinal study	United Kingdom	Yes	Adults	Deprivation
					Social networks
(113)	Longitudinal study	Finland	No	Adults	Social networks
(114)	Cross-sectional study	United Kingdom	No	Adults	Built environment
					Population structure
(115)	Cross-sectional study	United Kingdom	Yes	Adults	Built environment
(116)	Cross-sectional study	Ireland	Yes	Adults	Attitudinal factors toward vulnerable groups
					Social networks
(117)	Longitudinal study	United Kingdom	No	Adults	Built environment
					Deprivation
(118)	Cross-sectional study	Germany	Yes	Adults	Attitudinal factors toward vulnerable groups
(119)	Longitudinal study	Europe	No	Adults	Attitudinal factors toward vulnerable groups
(120)	Systematic review	Denmark Sweden Norway	Yes	Children	Attitudinal factors toward vulnerable groups
(121)	Cross-sectional study	Finland	Yes	Adults	Attitudinal factors toward vulnerable groups
(122)	Longitudinal study	Netherlands	No	Adults	Built environment
					Deprivation
					Population structure
(123)	Longitudinal study	United Kingdom	No	Adolescents	Built environment
					Pollution
					Security
					Social networks
(124)	Longitudinal study	United Kingdom	No	Children	Built environment
					Deprivation
					Pollution
(123)	Cross-sectional study	Germany	Yes	Adults	Attitudinal factors toward vulnerable groups
(125)	Cross-sectional study	Germany	Yes	Adolescents	Attitudinal factors toward vulnerable groups
					Social networks
(126)	Cross-sectional study	Germany	Yes	Adults	Attitudinal factors toward vulnerable groups
(127)	Longitudinal study	United Kingdom	No	Children	Built environment
					Deprivation
					Security
					Social networks
(128)	Longitudinal study	United Kingdom	No	Adults	Pollution
(129)	Cross-sectional study	Norway	Yes	Adults	Social networks
(130)	Cross-sectional study	United Kingdom	Yes	Adults	Attitudinal factors toward vulnerable groups
(131)	Longitudinal study	Czechia Netherlands France	No	Adults	Built environment
(132)	Cross-sectional study	Finland	No	Adults	Social networks
(133)	Cross-sectional study	Norway	Yes	Adolescents	Attitudinal factors toward vulnerable groups
					Social networks
(134)	Longitudinal study	Denmark	No	Adults	Built environment
(135)	Cross-sectional study	United Kingdom	Yes	Adults	Social networks
(136)	Cross-sectional study	Netherlands	No	Adults	Access to service
					Built environment
					Mobility
					Pollution
					Social networks
(137)	Cross-sectional study	United Kingdom	No	Adults	Access to service
					Built environment
					Security
(138)	Cross-sectional study	Finland	No	Adults	Attitudinal factors toward vulnerable groups
(139)	Cross-sectional study	Spain	Yes	Adults	Attitudinal factors toward vulnerable groups
(140)	Longitudinal study	United Kingdom	No	Adults	Mobility
					Policy
(141)	Longitudinal study	Ukraine	No	Children	Security
(142)	Cross-sectional study	United Kingdom	Yes	Adolescents	Security
					Social networks
(143)	Cross-sectional study	Georgia	No	Adults	Policy
(144)	Longitudinal study	United Kingdom	No	youth <19 years	Pollution
(145)	Cross-sectional study	Netherlands	No	Adults	Built environment
					Security
					Social networks
(146)	Cross-sectional study	Italy	No	Adults	Security
(147)	Cross-sectional study	Spain	No	Adults	Built environment
(148)	Cross-sectional study	Italy	Yes	Adults	Attitudinal factors toward vulnerable groups
(149)	Cross-sectional study	United Kingdom	No	Adolescents	Social networks
(150)	Cross-sectional study	United Kingdom Spain Lithuania Netherlands	No	Adults	Built environment
					Deprivation
					Social networks
(151)	Cross-sectional study	Spain	No	Adults	Attitudinal factors toward vulnerable groups
(152)	Qualitative study	Croatia	Yes	Adults	Attitudinal factors toward vulnerable groups
(153)	Longitudinal study	Sweden	No	Adults	Deprivation
					Population structures
(154)	Cross-sectional study	Austria	No	Adults	Mobility
(155)	Cross-sectional study	Germany	Yes	Adults	Attitudinal factors toward vulnerable groups
(156)	Longitudinal study	United Kingdom	No	Adults	Deprivation
					Social networks
(157)	Cross-sectional study	Italy	Yes	Adults	Attitudinal factors toward vulnerable groups
					Social networks
(158)	Cross-sectional study	Spain	No	Adults	Social networks
(159)	Cross-sectional study	Netherlands	No	Adults	Population structures
(160)	Systematic Review	Europe	No	Children	Pollution
(161)	Cross-sectional study	Germany	Yes	Adults	Attitudinal factors toward vulnerable groups
(162)	Cross-sectional study	Spain	Yes	Adults	Attitudinal factors toward vulnerable groups
					Deprivation
(163)	Cross-sectional study	Europe	No	Adults	Pollution
(164)	Longitudinal study	Netherlands	No	Adults	Social networks
(165)	Qualitative study	Georgia	Yes	Adults	Attitudinal factors toward vulnerable groups
					Social networks
(166)	Cross-sectional study	Netherlands	Yes	Adults	Attitudinal factors toward vulnerable groups
(167)	Mixed methods	United Kingdom	Yes	Adults	Social networks
(168)	Cross-sectional study	United Kingdom	Yes	Adults	Social networks
(169)	Systematic review	Norway	No	Adults	Social networks
(170)	Cross-sectional study	Germany	Yes	Adults	Attitudinal factors toward vulnerable groups
(171)	Cross-sectional study	Greece	Yes	Adults	Attitudinal factors toward vulnerable groups
					Macroeconomic environment
					Social networks
(172)	Cross-sectional study	Sweden	Yes	Adults	Attitudinal factors toward vulnerable groups
					Social networks
(173)	Longitudinal study	Czechia	Yes	Adults	Attitudinal factors toward vulnerable groups
(174)	Cross-sectional study	Macedonia	Yes	Adults	Attitudinal factors toward vulnerable groups
(175)	Longitudinal study	Netherlands	Yes	Adults	Attitudinal factors toward vulnerable groups
					Policy
(176)	Cross-sectional study	United Kingdom	No	Adults	Built environment
(177)	Cross-sectional study	United Kingdom	No	Adults	Security
(178)	Cross-sectional study	Ireland	Yes	Adults	Security
(179)	Qualitative study	Denmark	No	Adults	Built environment
(180)	Cross-sectional study	Norway	Yes	Adults	Social networks
(181)	Cross-sectional study	United Kingdom	Yes	Adults	Attitudinal factors toward vulnerable groups
(182)	Cross-sectional study	Spain Netherlands Lithuania United Kingdom	No	Adults	Built environment
(183)	Cross-sectional study	Spain Netherlands Lithuania United Kingdom	No	Adults	Built environment
(184)	Longitudinal study	Finland Sweden United Kingdom	Yes	Adolescents	Attitudinal factors toward vulnerable groups
					Social networks
(185)	Cross-sectional study	Iceland	No	Adolescents	Social networks
(186)	Cross-sectional study	Bosnia and Herzegovina	Yes	Adults	Security
(187)	Longitudinal study	Sweden	No	Adults	Built environment
(188)	Cross-sectional study	United Kingdom	No	Adults	Built environment
(189)	Cross-sectional study	United Kingdom	No	Adults	Deprivation social networks
(190)	Cross-sectional study	United Kingdom	Yes	Adults	Access to service
					Attitudinal factors toward vulnerable groups
(191)	Cross-sectional study	United Kingdom	Yes	Adults	Climate change social networks
(192)	Review	Germany	No	Adults	Pollution
(193)	Cross-sectional study	United Kingdom	No	Adults	Built environment
(194)	Longitudinal study	Poland	Yes	Adults	Attitudinal factors toward vulnerable groups
(195)	Longitudinal study	United Kingdom	No	Adults	Deprivation
(196)	Cross-sectional study	Macedonia	Yes	Adults	Attitudinal factors toward vulnerable groups

### 3.2. Characteristics of the included studies

The basic characteristics of the included studies are described in Table 1.

#### 3.2.1. Study population

Of the included studies, 95 (56%) of the studies focused on the general population, while 74 studies (44%) targeted high-risk populations. High-risk populations included ethical/racial minorities (*n* = 36), sexual/gender minorities (*n* = 14), people with comorbidities (*n* = 10), and other special populations (*n* = 14), such as pregnant women, persons who experienced war, caregivers, terrorist attack survivors, and disaster victims. Adults were mainly researched (*n* = 141, 83%), while children (*n* = 10*, 6%) and adolescents (*n* = 19*, 11%) were less often represented (*double count for one study).

In this Europe-focused review, populations from following countries were most often under investigation: The United Kingdom (*n* = 53), the Netherlands (*n* = 23), Spain and Sweden (*n* = 15 each), Germany (*n* = 13), and other (*n* = 42). Eight studies included pan-European datasets with data from more than eight European countries.

#### 3.2.2. Study outcomes

The most frequently reported outcomes were depressive symptoms/depression (*n* = 83, 49%), general mental health (*n* = 56, 33%), and anxiety disorders (*n* = 40, 23%). Other outcomes were well-being, psychological distress, post-traumatic stress disorder (PTSD), mood disorders, suicide/self-harm, schizophrenia, and “other” which includes health service use, any psychiatric disorder, substance abuse, intellectual disability, resilience, and loneliness. However, the study outcomes are independent of study design and statistical significance, but only based on being mentioned as such.

#### 3.2.3. Study types

About 60% (*n* = 99) of the studies were cross-sectional studies, 27% (*n* = 46) were longitudinal studies, the remaining 24 studies (14%) were systematic reviews, qualitative studies, reviews, experimental studies, mixed methods studies, ecological studies, meta-analysis, and case–control studies.

### 3.3. Indicators and indicator categories

Of the included 169 studies, 53 indicators were identified and grouped conceptually into 13 indicator categories for mental health. These indicator categories again were assigned to either the physical or social environmental domain of community indicators. In the domain of the social environment, eight indicator categories with 36 indicators are described. In the domain of the physical environment, 17 indicators were grouped into five indicator categories. The most commonly investigated indicator categories are social networks (87 times), attitudinal factors toward vulnerable groups (76 times), built environment (56 times), deprivation (40 times), and security (34 times). Details on the division of the indicators regarding the two domains can be found in Table 2.

**Table 2:** Community indicator categories and single indicators.

Indicator categories and corresponding indicators		Occurrence(s)
Domain: Social environment		271
1. Social networks		87
	Social capital	23
	Social support	20
	Social cohesion	18
	Trust	14
	Community connectedness	11
	Peer support	1
2. Attitudinal factors toward vulnerable groups		76
	Discrimination	55
	Minority stress	12
	Stigma	7
	Racism	2
3. Deprivation		41
	Neighborhood deprivation	19
	Socioeconomic deprivation	18
	Average house price	1
	Average income	1
	Income inequality	1
	Percentage of low-income earners	1
4. Security		34
	Crimes	14
	Perceived safety	7
	Victimization	6
	Violence	5
	Conflict	1
	Right-wing authoritarianism	1
5. Population structures		16
	Ethnic density	6
	Population density	3
	Family structure	2
	Household structure	1
	Move-outs per residents	1
	Population structure	1
	Social gradient	1
	Single households	1
6. Access to services		8
	Accessibility	8
7. Macroeconomic environment*		5
	Financial crisis	5
8. Policy*		4
	Alcohol availability, marketing, and pricing	1
	Migrant integration on national level	1
	Social conditions in host country	1
	Transportation policy change	1
Domain: Physical environment		96
9. Built environment		56
	Green/blue space	25
	Built density (urban/rural)	15
	Housing (access to and quality of)	8
	Built neighborhood environment	5
	Attractiveness	2
	Indoor sunlight exposure	1
10. Pollution*		24
	Air pollution	13
	Noise pollution	10
	Exposure to toxins	1
11. Mobility*		10
	Transportation/public transit	6
	Walkability	3
	Bike infrastructure	1
12. Presence of resources*		4
	Presence of facilities in the neighborhood (stores, schools, health facilities, green space)	1
	Presence of higher-level facilities	1
	Presence of physical facilities (Elder and health facilities, nursing homes and culture/sports/recreation facilities among others)	1
	Presence of social resources (Sports/cultural, health support associations, educative associations, and local/professional groups)	1
13. Climate change*		2
	Natural disaster	2

Community indicator categories: marked blue with bold letters and numbered; Indicators: not numbered; * new indicator categories.

#### 3.3.1. Composite indicators

Composite indicators combine a set of individual indicators into a single index (197). To account for the distinct aspects of the neighborhood, 11 studies used composite indices/indicators to depict either the social or physical environment, or both in the context of mental health. The composite indicators measure neighborhood quality (137), neighborhood composition (51), neighborhood usability (60), social disadvantage (32, 33), community resources accessibility index (61), community-level alcohol environment (B (143).), ecological assets (45), neighborhood characteristics (91), and post-migration living difficulties (172). Among these composite indicators, the most frequently used were “access to services” (*n* = 4) and “neighborhood deprivation” (*n* = 3).

#### 3.3.2. Indicator categories by outcomes

Regarding general mental health, social networks (*n* = 25), discrimination (*n* = 24), built environment (*n* = 18), deprivation (*n* = 15), and violence (*n* = 6) were most studied. For depression, the most common indicators were social networks (*n* = 30), discrimination (*n* = 28), built environment (*n* = 23), deprivation (*n* = 13), pollution (*n* = 10), and violence (*n* = 9). Similarly, regarding anxiety disorders, the most frequently investigated indicator categories were discrimination (*n* = 17), social networks (*n* = 13), violence (*n* = 6), deprivation (*n* = 5), built environment (*n* = 5), and pollution (*n* = 3). The other mental health conditions were most investigated regarding social networks (*n* = 13), built environment (*n* = 7) and violence (*n* = 7), discrimination (*n* = 4), and urbanization and policy (each *n* = 3).

#### 3.3.3. Indicator categories by population

The identified indicators in the included studies vary by population. The most frequently investigated categories in the general population were social networks (*n* = 41), built environment (*n* = 38), deprivation (*n* = 19), security (*n* = 16), and pollution (*n* = 15), while in the high-risk-population attitudinal factors toward vulnerable groups (*n* = 51) and social network (*n* = 31) were by far the most important indicator categories (see Table 3).

**Table 3:** Number of occurrences of the indicator categories by risk and age group displayed in different blue shades according to frequency.

Indicator categories	Population				
	General	High-risk	Children (<13 years)	Adolescents (10–19 years)	Adults (>18 years)
Built environment	38	9	5	4	38
Climate change	1	0	0	0	1
Mobility	6	0	0	0	6
Pollution	15	0	5	2	9
Presence of resources	2	0	0	0	2
Access to service	5	3	0	0	8
Attitudinal factors toward vulnerable groups	7	51	1	6	51
Deprivation	19	7	3	3	20
Macroeconomic environment	4	1	0	0	5
Security	16	8	2	6	16
Policy	1	3	0	0	4
Population structures	7	1	0	1	7
Social networks	41	31	1	13	57

#### 3.3.4. Indicator categories by special groups

Attitudinal factors toward vulnerable groups and social networks are most commonly researched in ethnical/racial and sexual/gender minorities. For people with comorbidities, attitudinal factors toward vulnerable groups were frequently investigated. “Comorbid patients” where defined as those with diagnosed epilepsy, HIV, schizophrenia, depression, mental health problems, borderline, intellectual impairment, anxiety/somatoform disorder, or obesity. In this population group, attitudinal factors toward vulnerable groups were most researched. Social networks were the most often investigated indicator category in “Other population” such as adults who experienced war, caregivers, pregnant women, survivors of terrorist attacks, disaster victims, mothers, and persons after bereavement (see Table 4).

**Table 4:** Number of occurrences of the indicator categories in the identified studies by special groups displayed in different blue shades according to frequency.

Community indicator categories	Population			
	Comorbid population	Ethnic/Racial minority	Sexual/Gender minority	Other
Built environment	2	6	0	1
Climate change	0	0	0	0
Mobility	0	0	0	0
Pollution	0	0	0	0
Presence of resources	0	0	0	0
Access to service	2	0	1	0
Attitudinal factors toward vulnerable groups	5	32	12	2
Deprivation	1	5	0	0
Macroeconomic environment	0	1	0	0
Security	1	1	3	3
Policy	0	2	1	0
Population structures	1	0	0	0
Social networks	1	16	5	9

## 4. Discussion

### 4.1. Summary of main findings

With this scoping review, we aim at providing a framework for relevant community indicators and public mental health measures in general and high-risk populations with a focus on Europe. We identified 53 community indicators that were clustered into 13 indicator categories, divided into the domains of physical and social environment. In comparison to the physical environment, the domain of the social environment has been studied more frequently. Overall, the indicator categories social networks, attitudinal factors toward vulnerable groups, and the built environment were the most frequently discussed topics. Pollution, mobility, presence of resources, and climate change, as well as population structure, macroeconomic environment, and policies were highlighted as new in terms of not yet being part of or under consideration for mental health surveillance. Many of the identified indicators and indicator categories are not yet part of existing monitoring systems but complement the picture of those indicators that should be included in the biopsychosocial models of disease in the future.

The understanding of mental health as a multi-faceted concept is translated into the latest WHO’s Comprehensive Mental Health Action Plan’s recommendation to gather “at least a core set of mental health indicators” every 2 years in a national Mental Health Surveillance (MHS) (198). There, environmental factors such as national policies, social protection, living standards, working conditions, and community social support are addressed and call for a multi-sectorial approach in the management and prevention of mental health disorders. Such indicator sets that cover a wide range of personal, social, and physical aspects are valuable to communities to monitor the community’s characteristics and needs, and to act appropriately with adequate mental health promotion and prevention strategies (199). Despite the importance of community-level indicators, the German Robert Koch Institute found them to be underrepresented in current surveillances and communicated it as a gap to be filled (200).

The community-level indicators range in a wide field of important areas and were structured by identifying the 13 superordinate indicator categories. As the indicators are extracted from exclusively scientific studies, we cannot make statements whether routinely monitored data are widely available. As most indicators were extracted from different studies, we cannot assume that there are existing databases for the individual indicator nor that there is a consensus on how it is operationalized.

The International classification of functioning, disability, and health (ICF) endorsed by the World Health Assembly for the international use (WHO) in 2001, is an important framework for the description of health and health related status of an individual in its specific context with a unified and standard language (201). With the adoption of the ICF, the understanding of functioning and disability has changed fundamentally (202). Individuals are not seen as unrelated entities anymore, but the individual’s functioning and health condition seen as in constant interaction with contextual factors such as social surroundings (202). In this classification, context factors are divided into personal and environmental factors, where the latter form the physical, social, and attitudinal environment in which people live and conduct their lives (201, 203). *While the social and attitudinal environments are widely discussed in the identified literature, the categories of the physical environment are less well investigated.*

The concept of environmental factors is generally based on the sociophysical context, which is understood as the observation that health is affected by the social and physical environment. The term sociophysical context is often not clearly defined in literature but shows overlaps with the concepts of community and neighborhood. In a broader sense, the place of residence with its specific environment affects the community members´ health condition through either direct (e.g., air pollution) or indirect factors (e.g., access to health care and social services) (136). Environmental factors could explain the repeated observation of health outcome differences across geographic areas (204). The growing interest in the contributing aspects of the community and neighborhood on the health status and lives of its residents urges the academic community to clearly define these terms in their research (205).

The domain of the social environment, especially the indicator category of social network, is well represented in this scoping review. The social network, which in various operationalizations comprises social capital, social cohesion, trust, community connectedness, peer and social support, is increasingly regarded as relevant (136). These indicators are believed to be mental health-related neighborhood/community factors, although the observed associations in the literature may be confounded by genetic or other environmental factors (206).

The lack of consensus on comparable measures of the social environment and the difficulties in obtaining them, makes the comparisons of the social environment complicated (204). For example, the definition and measurement of social capital shows great heterogeneity, ranging from individual to neighborhood-level measures. To obtain valid measures of social capital, proxy indicators such as voter turnout (81), or survey data (41, 64, 164, 191) are commonly used. The indicator category of social networks is also listed in the RKI scoping review (200) and in the ICF (201) expressing its relevance.

The indicators of discrimination, minority stress, stigma and racism were assigned to the superordinate topic “attitudinal factors toward vulnerable people” and represent the attitudinal environment. In the ICF, attitudinal factors are described as observable consequences of customs, ideologies, values, and norms influencing both individual behavior and social life at all levels (201, 203). This affects individuals in their personal and professional lives, as well as societal attitudinal factors and social norms. In the identified studies, attitudinal factors toward vulnerable groups were most studied in high-risk groups, mainly in ethnic/racial and sexual/gender minorities. Discrimination is associated with a higher cumulative risk of negative long-term effects on mental health (207). The RKI identified stigma, self-stigma and anti-stigma movements, discrimination, discrimination due to mental health problems, and perceived legitimacy of discrimination of people with mental disorders as six relevant indicators for a future national MHS (200). This is of enormous importance as heterogeneous communities are advised to create an inclusive and supportive environment to prevent mental illness in the first place (207). This goes in line with the recommendation provided in the ECCM.

In the prevention and health promotion oriented ECCM (11), the newly introduced categories in the domain of community resources and policies include the creation of supportive environments, building healthy public policies and strengthening community action. The identified indicators can support the ECCM by providing measures to indicate the direction of action but also enrich the ECCM toward an Extended Chronic *Mental* Care Model. As indicators rely on continuous good quality data, a “broadly-based information system to include community data beyond the health care system” is needed and also suggested by ECCM (11). Routinely collected data can provide an integrated picture to fully understand the communities´ context and needs to inform policy makers, municipalities, and stakeholders. It is worth noticing that many community indicators in the identified literature were assessed based on primary data. Routinely collected data on the physical and social environment is often not available, which imposes a barrier to monitoring.

Factors in the physical environment that had been proven to impact people’s health include harmful substances (e.g., air pollution), access to health-related resources and the built environment (204, 208). The indicator category of the built environment was most frequently discussed in the reviewed literature (*n* = 41). It comprises the indicators: built neighborhood environment, access to and quality of housing, green and blue space, attractiveness, and indoor sunlight opportunity. Of these, green and blue spaces were studied most frequently and their association with mental health is getting more attention. Still, the physical environment domain is less frequently researched compared to the social environment in the context of mental health. It was neglected for a long time but meanwhile there is growing evidence for its contribution to mental health (209).

Several studies documented the effects of elements of the physical environment (e.g., exposure to harmful substances in the air/air pollution) on somatic diseases, especially cardiovascular and respiratory morbidity (204, 210–213). Specific disease-related mechanisms had been identified by which these exposures affect inflammatory, autonomic, and vascular processes (204, 210, 213). These processes can also influence mental health and the initiation of mental disease (214, 215).

The built environment can influence mental health directly or indirectly by affecting psychosocial and behavioral processes (209). Such processes that are known to be linked to mental health are for example personal control (e.g., over noise and crowded homes), social support (e.g., great distance and residence and high-traffic volume streets is associated with reduced social interaction) as well as restoration and recovery from fatigue and stress (e.g., by the presence of natural elements). These hypotheses and the growing amount of literature underpinning them stress the need to include physical environment indicators in population mental health surveillances. Also, the ICF considers the built environment in the chapter “Natural environment and human-made changes to environment.” Among the methodological challenges to estimate the association between the physical environment and mental health are the self-selection of individuals into settings, inadequate environmental measurements, poor exposure assessment and over-reliance on self-reported indicators (209).

In this review, the distribution of community-level indicators and the representation of populations varied among the identified studies. While high-risk populations were commonly studied under the aspect of attitudinal factors toward vulnerable groups and social networks, the indicator categories of the general population were distributed more evenly between social networks, built environment, security, deprivation, and pollution. Adults were more frequently studied than children and adolescents (*n* = 141 vs. *n* = 28, respectively). For the age group <19 years, there are only few studies available, which could be regarded as a major “gap” as the average onset of mental health disorders is at the age of 14.5 years (216). Primary prevention and early intervention programs can alter the course of mental health disorders and improve outcomes at the same time (217, 218). Mental health promotion and prevention achieves its optimal benefits when young people are targeted at the time of or even before onset of mental health problems (216).

In high-risk populations, minority groups, followed by patients with comorbidities, were most frequently studied (*n* = 85 and *n* = 13, respectively). Despite the known association between physical disease and depression, this did not translate into the amount of literature identified. This lack is surprising, as especially in the development and severity of cardiovascular disease, social (219) and environmental (220) aspects are known risk factors. Also, only a few studies on post-partum women, another risk group for the development of mental disorders, were included despite a prevalence of up to 15% of depression in this group (221). The exclusion of post-partum women may be due to the strict exclusion criteria as this group is mainly investigated in a clinical setting.

Many of the identified community indicators are not included in existing surveillance and monitoring systems, possibly because they are either novel, continue to be unrecognized, or face operationalization challenges. Up to now, the physical environment was less recognized as relevant for public mental health. In particular, climate change, and pollution is receiving more attention due to the collective crisis awareness. In this review, the focus is on the community setting, which results in only two studies on climate change and mental health despite rapidly growing body of literature (222). Nonetheless, we emphasize that climate change and the associated climate emergency and its relation to mental health should be picked up in future research. Climate change will continue to contribute to more natural disasters in the future which may impact people’s wellbeing to a large degree and affect a greater proportion of the population (223). Climate change and pollution must be addressed on a community level by low-threshold interventions (e.g., by planting more trees against heat, noise, and pollution) but also on a national and global level.

Given the constant interaction and exposure of the individual to features of the social and physical environment, even minor changes can have a significant impact on population health. Communities can act as facilitators for mental health promotion and prevention and may therefore significantly support the efforts of the mental health care sector.

### 4.2. Limitations and future research

Applying state-of-the-art methodology, this scoping review also comes with its limitations: First, the terms “community” and “neighborhood” are often insufficiently defined in studies and therefore the authors had to make explicit decisions about what a person perceives as their area of residence. In this review, we refer to the community as people living in the same geographical area, which is clearly just one core element of a community. As most patients with mental health problems are treated in primary care settings and one central pillar of the Chronic Care Model concept are community resources, we were interested in seeing which community factors can add to the management of mental health problems. As we focus on geographical communities, we neglected communities that were beyond spatial boundaries such as online communities. Hence, we also explicitly excluded studies with a digital focus, which may in turn offer additional factors that might have been excluded.

Second, scoping reviews are not intended to provide in-depth information on the included studies, nor do they provide information on the relationship or correlation between each indicator and mental health. As we report the frequency of published papers with the mentioned indicators, a publication and researcher bias should be taken into account. And it is very possible that there is more data available or accessible for certain indicators than for others without meaning that the frequency of investigations reflects the importance or relevance of an indicator. No methodological quality appraisal of the included studies was conducted, which limits the implications for practice. Further studies should conduct a more extensive research on the identified indicator categories. With this scoping review, we point to community indicators, which have been of interest in previous studies and direct us to new fields for future research. Within the scope of spin-off projects, it will be necessary to hone in on the key domains, identify standardized tools within these areas, and evaluate their applicability, implementation, value, and usage within the context of community mental health approaches.

Third, due to the strict exclusion criteria, especially regarding the location as we were focusing only on the European context, some indicators could not (e.g., disasters and terrorism) or just to a limited degree (e.g., climate change) be represented in the final synthesis.

Fourth, the literature search was limited to three databases, so we could probably not cover the entire breadth of community settings.

This scoping review provides the first collection of community-level indicators that may influence public mental health. More research is needed to evaluate the operationalization, contribution and the prognostic relevance of each indicator on mental health and should be routinely included in mental health surveillance systems as cofounders. Further, relevant community-level indicators should be considered in the management and prevention of mental disorders.

### 4.3. Conclusion

This scoping review covers a comprehensive set of community indicators, some of which are already well known as contributing factors to mental health, and others whose importance has recently gained more attention and may reveal latest trends in key areas. It complements the picture of community factors that should be represented in the biopsychosocial models of disease, and should also be routinely collected in surveillance systems to investigate their importance and role as a confounding factor. Future research should, on the one hand focus more on children and adolescents as this is the time when mental health promotion and prevention should start, and on the other hand on emerging new fields such as climate change.

## Data availability statement

The original contributions presented in the study are included in the article, further inquiries can be directed to the corresponding author.

## POKAL-group

The following doctoral students are as well members of the POKAL-Group: Jochen Vukas, Puya Younesi, Feyza Gökce, Victoria von Schrottenberg, Petra Schönweger, Hannah Schillock, Jonas Raub, Philipp Reindl-Spanner, Lisa Hattenkofer, Lukas Kaupe, Carolin Haas, Julia Eder, Vita Brisnik, Constantin Brand and Katharina Biersack.

## Author contributions

PS and CJ-S defined and developed the study question, protocol, and search strategy. PS, A-FM, KB, PR-S, and MK performed title, abstract, and full-text screening, as well as data extraction. Attachments and figures were composed by PS and A-FM. The manuscript was commented by MK, KB, A-FM, BP, JG, PH, HK, and CD-J. The manuscript was finally drafted by PS and finalized by PS and CJ-S. All authors contributed to the article and approved the submitted version.

## Funding

The Research Training Group “PrediktOren und Klinische Ergebnisse bei depressiven ErkrAnkungen in der hausärztLichen Versorgung” (POKAL, DFG-GRK 2621; Predictors and Clinical Outcomes of Depressive Disorders in Primary Care) POKAL is a member of the German Research Foundation (DFG) and has developed the comprehensive qualification concept that aims to effectively support both health care providers and stakeholders and improve the future treatment of depression in primary care. The focus is on depression as the most common diagnosis.

## Conflict of interest

The authors declare that the research was conducted in the absence of any commercial or financial relationships that could be construed as a potential conflict of interest.

## Publisher’s note

All claims expressed in this article are solely those of the authors and do not necessarily represent those of their affiliated organizations, or those of the publisher, the editors and the reviewers. Any product that may be evaluated in this article, or claim that may be made by its manufacturer, is not guaranteed or endorsed by the publisher.
