# Supplementary material for: Community indicators for mental health in Europe: a scoping review
Source: Front Public Health. 2023 Jul 19;11:1188494. doi: 10.3389/fpubh.2023.1188494 (PMC10396773; doi:10.3389/fpubh.2023.1188494)
Supplement: Supplementary file 1 [file Table_1.DOCX]

Supplementary Material

**Community Indicators for Mental Health in Europe: A Scoping Review**

**Schoenweger Petra*^1,2^, Kirschneck Michaela^1,2^, Biersack Katharina^3^, Di Meo Anna-Francesca^1,2^, Reindl-Spanner Philip^4^, Prommegger Barbara^4^, Ditzen-Janotta Claudia^1,2^, Henningsen Peter^3^, Krcmar Helmut^4^, Gensichen Jochen^5^, Jung-Sievers Caroline^1,2^**

***Correspondence:**

Petra Schoenweger

petra.schoenweger@ibe.med.uni-muenchen.de

**Supplementary material- Table 1** - PCC framework of scoping review

| **Category** | **Determinants** | **Included topics** |
| --- | --- | --- |
| Population | Communities | Members in a community, towns, districts, neighborhoods |
| Concept | Community-level indicators | Community factors, community-level indicators,  risk/protective factors, neighborhood factors, environment characteristics  Public health surveillance  Public health monitoring |
| Context | Public mental health in towns, communities and neighborhoods, health promotion and prevention, health services  European countries | *Public mental health:*  (Depression, mental health, anxiety)  *Built-/physical environment* (walkability, greenness, service facilities, health care structures, transportation/public transit, accessibility, noise, and air pollution)  *Social/socioeconomic environment* (community connectedness, peer support, social capital, safety, trust, crimes, community disadvantage, discrimination) |
| Study type | Primary studies (quantitative and qualitative), reviews and meta-analysis will be included. |  |

**Supplementary material- Table 2** - Pubmed Search Syntax

| **Search Syntax- Pubmed** |
| --- |
| ((((("community disadvantage*"  OR "discrimination"  OR "crime*"  OR "trust"  OR "safety"  OR "social capital"  OR "peer support"  OR "community connect*"  OR "air pollut*"  OR "noise pollut*"  OR "accessibility"  OR "public transit"  OR "public transport*"  OR "health care structur*"  OR "service facilit*"  OR "green*"  OR "walkab*"  OR "surveillance"  OR "Population surveillance"  OR "epidemiological monitoring"  OR "Public health surveillance"  OR "residential risk factor*"  OR "residential factor*"  OR "residential characteristic*"  OR "environment determinant*"  OR "environment risk factor*"  OR "environment characteristic*"  OR "environment factor*"  OR "environment indicator*"  OR "community level characteristic*"  OR "community level risk factor*"  OR "community level factor*"  OR "community level indicator*"  OR "community determinant*"  OR "community characteristic*"  OR "community risk factor*"  OR "community factor*"  OR "community indicator*"  OR "neighborhood determinant*"  OR "neighborhood risk factor*"  OR "neighborhood characteristic*"  OR "neighborhood factor*"  OR "neighborhood indicator*"  OR "neighbourhood determinant*"  OR "neighbourhood risk factor*"  OR "neighbourhood characteristic*"  OR "neighbourhood factor*"  OR "neighbourhood indicator*")  AND ("mental health"  OR "mental health"[MeSH Terms]  OR "depressive disorder*"  OR "mental disorder*"  OR "depression"  OR "anxiety"))  NOT ("sars cov 2"[MeSH Terms]  OR "sars cov 2"[All Fields]  OR "covid"[All Fields]  OR "covid 19"[MeSH Terms]  OR "covid 19"[All Fields]))  AND ((y_10[Filter])  AND (meta-analysis[Filter]  OR observationalstudy[Filter]  OR randomizedcontrolledtrial[Filter]  OR review[Filter]  OR systematicreview[Filter])  AND (english[Filter]  OR german[Filter])))  NOT (((((America*)  OR (Australia*))  OR (US*))  OR (Asia))  OR (Africa)))  NOT (((drug*)  OR (pharmacologic*))  OR (medication)) |

If not mentioned otherwise in square brackets following the search term, all search terms were searched for in the field [Title/Abstract].

**Supplementary material- Table 3** - Overview of included studies

| **Author/ Year** | **Study Type** | **Region** | **Population** | | **Community Indicator Categories** |
| --- | --- | --- | --- | --- | --- |
|  |  |  | High Risk | Age Group |  |
| Abebe et al., 2014 | Systematic review | Norway | yes | Adults | Attitudinal factors towards vulnerable groups  Built environment  Social networks |
| Adriaanse et al., 2014 | Cross-sectional study | Netherlands | yes | Adolescents | Attitudinal factors towards vulnerable groups Built environment  Deprivation Social networks |
| Adriaanse et al., 2016 | Cross-sectional study | Netherlands | yes | Adolescents | Attitudinal factors towards vulnerable groups Built environment  Deprivation |
| Ahnquist et al., 2012 | Review | Sweden | no | Adults | Social networks |
| Aichberger et al., 2015 | Cross-sectional study | Germany | yes | Adults | Attitudinal factors towards vulnerable groups |
| Almquist et al., 2014 | Cross-sectional study | Sweden | no | Adolescents | Social networks |
| Astell-Burt et al., 2014 | Longitudinal study | United Kingdom | no | Adults | Built environment |
| Bailey et al., 2021 | Qualitative study | United Kingdom | no | Adults | Security |
| Bakic et al., 2019 | Longitudinal study | Croatia | no | Adults | Climate change  Social networks |
| Bakic et al., 2021 | Case-Control | Croatia | yes | Adults | Social networks |
| Bamford et al., 2021 | Cross-sectional study | United Kingdom | yes | Adults | Deprivation  Social networks |
| Baranyi et al., 2019 | Longitudinal study | Europe | no | Adults | Access to services  Pollution |
| Barratt et al., 2015 | Qualitative study | United Kingdom | no | Adults | Built environment |
| Barriuso-Lapresa et al., 2012 | Cross-sectional study | Spain | no | Children | Deprivation |
| Bastos et al., 2015 | Cross-sectional study | Portugal | no | Adults | Access to services  Presence of resources |
| Borsch et al., 2019 | Systematic review | Denmark Finland Norway  Sweden | yes | Adults | Attitudinal factors towards vulnerable groups Social networks |
| Botchkovar et al., 2018 | Cross-sectional study | Ukraine | no | Adults | Security |
| Castaneda et al., 2015 | Cross-sectional study | Finland | yes | Adults | Attitudinal factors towards vulnerable groups |
| Cecil et al., 2014 | Cross-sectional study | United Kingdom | yes | Adolescents | Security |
| Chaves et al., 2018 | Cross-sectional study | Spain Europe | no | Adults | Macroeconomic environment |
| Cramer et al., 2013 | Ecological study | Germany | yes | Adults | Built environment  Population structure |
| Cramm et al., 2013 | Cross-sectional study | Netherlands | no | Adults | Social networks |
| Curtis et al., 2013 | Longitudinal study | United Kingdom | no | Adults | Deprivation |
| Dabbagh et al., 2012 | Cross-sectional study | United Kingdom | yes | Adolescents | Attitudinal factors towards vulnerable groups |
| De Freitas et al., 2018 | Meta- analysis | Europe | yes | Adults | Attitudinal factors towards vulnerable groups |
| De Vries et al., 2013 | Cross-sectional study | Netherlands | no | Adults | Built environment Social networks |
| Delaruelle et al., 2021 | Cross-sectional study | Europe | yes | Adolescents | Social networks |
| Derdikman‐Eiron et al., 2013 | Longitudinal study | Norway | no | Adolescents | Social networks |
| Dewaele et al., 2014 | Cross-sectional study | Belgium | yes | Adults | Attitudinal factors towards vulnerable groups Social networks |
| Domènech-Abella et al., 2020 | Cross-sectional study | Finland Poland Spain | no | Adults | Built environment Mobility Social networks |
| Donisi et al., 2013 | Ecological study | Italy | yes | Adults | Access to services  Presence of resources |
| Economou et al., 2018 | Cross-sectional study | Cyprus | no | Adults | Macroeconomic environment Social networks |
| Economou et al., 2014 | Cross-sectional study | Greece | no | Adults | Macroeconomic environment  Social networks |
| Elliott et al., 2014b | Mixed methods | United Kingdom | no | Adults | Social networks |
| Engemann et al., 2019 | Longitudinal study | Denmark | no | Adults | Built environment |
| Engemann et al., 2020 | Longitudinal study | Denmark | no | Children | Built environment |
| Essers et al., 2022 | Longitudinal study | Spain Netherlands | no | Children | Pollution |
| Ettema & Schekkerman, 2016 | Cross-sectional study | Netherlands | no | Adults | Access to service  Built environment  Mobility  Presence of resources Population structures Social networks |
| Finegan et al., 2020 | Longitudinal study | United Kingdom | no | Adults | Deprivation |
| Fone et al., 2014 | Longitudinal study | United Kingdom | no | Adults | Social networks |
| Forsman et al., 2012 | Cross-sectional study | Finland Sweden | no | Adults | Social networks |
| Foy et al., 2019 | Longitudinal study | United Kingdom | yes | Adults | Access to services  Presence of resources |
| Freitas et al., 2016 | Cross-sectional study | Portugal | yes | Adolescents | Attitudinal factors towards vulnerable groups |
| Friborg et al., 2017 | Cross-sectional study | Norway | yes | Adults | Attitudinal factors towards vulnerable groups |
| Gaum et al., 2019 | Longitudinal study | Germany | no | Adults | Pollution |
| Generaal et al., 2019 | Cross-sectional study | Netherlands | no | Adults | Built environment  Deprivation  Pollution  Security  Social networks |
| Generaal et al., 2019 | Cross-sectional study | Netherlands | no | Adults | Built environment Deprivation  Pollution  Security Social networks |
| Gnan et al., 2019 | Cross-sectional study | United Kingdom | yes | Adults | Attitudinal factors towards vulnerable groups Security  Social networks |
| Gonzales-Inca 2022 | Longitudinal study | Finland | no | Adults | Built environment |
| Gubbels et al., 2016 | Longitudinal study | Netherlands | no | Adolescents | Built environment |
| Hackett et al., 2019 | Cross-sectional study | United Kingdom | no | Adults | Attitudinal factors towards vulnerable groups |
| Hamano et al., 2018 | Longitudinal study | Sweden | no | Adults | Deprivation Social networks |
| Hansen & Sørlie, 2012 | Cross-sectional study | Norway | yes | Adults | Attitudinal factors towards vulnerable groups |
| Hatch et al., 2016 | Cross-sectional study | United Kingdom | yes | Adults | Attitudinal factors towards vulnerable groups |
| Helsen et al., 2021 | Cross-sectional study | Netherlands | yes | Adults | Attitudinal factors towards vulnerable groups Social networks |
| Hüfner et al., 2020 | Cross-sectional study | Austria | yes | Adults | Built environment |
| Igde et al., 2019 | Systematic review | Germany | yes | Adults | Attitudinal factors towards vulnerable groups |
| Ikram et al., 2015 | Cross-sectional study | Netherlands | yes | Adults | Attitudinal factors towards vulnerable groups |
| Infurna et al., 2015 | Cross-sectional study | Italy | no | Adults | Social networks |
| Jackson et al., 2019 | Longitudinal study | United Kingdom | yes | Adults | Attitudinal factors towards vulnerable groups |
| Junghans & Jones, 2007 | Cross-sectional study | United Kingdom | no | Adults | Social networks Security |
| Jonsson et al., 2018 | Cross-sectional study | United Kingdom | no | Adolescents | Deprivation  Population structure Security |
| Källström et al., 2020 | Longitudinal study | Sweden | no | Adults | Security |
| Kendrick et al., 2012 | Cross-sectional study | Sweden | no | Adolescents | Social networks |
| Klaperski et al., 2019 | Other | Germany | no | Adults | Built environment |
| Knott et al., 2018 | Longitudinal study | United Kingdom | no | Adults | Mobility |
| Kreski et al., 2018 | Cross-sectional study | France | yes | Adults | Security |
| Kritsotakis et al., 2013 | Longitudinal study | Greece | yes | Adults | Social networks |
| Kunst et al., 2013 | Cross-sectional study | Netherlands | no | Adults | Security |
| Lafferty et al., 2022(A. et al., 2022) | Longitudinal study | United Kingdom | yes | Adults | Deprivation |
| Lakeman et al., 2012 | Qualitative study | Ireland | no | Adults | Attitudinal factors towards vulnerable groups |
| Landstedt et al., 2016 | Longitudinal study | Sweden | no | Adults | Social networks |
| Lanfredi et al., 2015 | Cross-sectional study | Europe | no | Adults | Attitudinal factors towards vulnerable groups Social networks |
| Latham et al., 2021 | Longitudinal study | United Kingdom | no | Children | Pollution |
| Lecerof et al., 2016 | Cross-sectional study | Sweden | yes | Adults | Built environment Deprivation  Social networks |
| Lee et al., 2014 | Cross-sectional study | Romania Bulgaria | yes | Adults | Attitudinal factors towards vulnerable groups |
| Levecque & Van Rossem, 2015 | Cross-sectional study | Europe | yes | Adults | Attitudinal factors towards vulnerable groups Policy |
| Lindström & Rosvall, 2012 | Cross-sectional study | Sweden | no | Adults | Social networks Deprivation |
| Lindström & Giordano, 2016 | Longitudinal study | United Kingdom | no | Adults | Social networks Macroeconomic environment |
| Llorente et al., 2018 | Cross-sectional study | Spain | no | Adults | Built environment Population structure |
| Magallares et al., 2014 | Cross-sectional study | Spain | no | Adults | Attitudinal factors towards vulnerable groups |
| Magallares et al., 2017 | Cross-sectional study | Spain | yes | Adults | Attitudinal factors towards vulnerable groups |
| Mangalore & Knapp, 2012 | Longitudinal study | United Kingdom | yes | Adults | Deprivation  Social networks |
| Markkula et al., 2016 | Longitudinal study | Finland | no | Adults | Social networks |
| Maxwell et al., 2021 | Cross-sectional study | United Kingdom | no | Adults | Built environment Population structure |
| McEachan et al., 2016 | Cross-sectional study | United Kingdom | yes | Adults | Built environment |
| McGorrian et al., 2013 | Cross-sectional study | Ireland | yes | Adults | Attitudinal factors towards vulnerable groups Social networks |
| Mears et al., 2020 | Longitudinal study | United Kingdom | no | Adults | Built environment Deprivation |
| Mewes et al., 2015 | Cross-sectional study | Germany | yes | Adults | Attitudinal factors towards vulnerable groups |
| Missinne & Bracke, 2012 | Longitudinal study | Europe | no | Adults | Attitudinal factors towards vulnerable groups |
| Mock-Munoz 2019 | Systematic review | Denmark Sweden Norway | yes | Children | Attitudinal factors towards vulnerable groups |
| Mölsä et al., 2017 | Cross-sectional study | Finland | yes | Adults | Attitudinal factors towards vulnerable groups |
| Motoc et al., 2019 | Longitudinal study | Netherlands | no | Adults | Built environment  Deprivation  Population structure |
| Mueller et al., 2019 | Longitudinal study | United Kingdom | no | Adolescents | Built environment Pollution  Security Social networks |
| Mueller & Flouri, 2020 | Longitudinal study | United Kingdom | no | Children | Built environment  Deprivation Pollution |
| Mueller et al., 2019 | Cross-sectional study | Germany | yes | Adults | Attitudinal factors towards vulnerable groups |
| Müller et al., 2021 | Cross-sectional study | Germany | yes | Adolescents | Attitudinal factors towards vulnerable groups Social networks |
| Nesterko et al., 2014 | Cross-sectional study | Germany | yes | Adults | Attitudinal factors towards vulnerable groups |
| Newbury et al., 2016 | Longitudinal study | United Kingdom | no | Children | Built environment Deprivation  Security Social networks |
| Newbury et al., 2021 | Longitudinal study | United Kingdom | no | Adults | Pollution |
| Nilsen et al., 2019 | Cross-sectional study | Norway | yes | Adults | Social networks |
| Nimmo‐Smith et al., 2016 | Cross-sectional study | United Kingdom | yes | Adults | Attitudinal factors towards vulnerable groups |
| Noordzij et al., 2021 | Longitudinal study | Czechia Netherlands France | no | Adults | Built environment |
| Nyqvist et al., 2013 | Cross-sectional study | Finland | no | Adults | Social networks |
| Oppedal & Idsoe, 2015 | Cross-sectional study | Norway | yes | Adolescents | Attitudinal factors towards vulnerable groups  Social networks |
| Østergaard et al., 2013 | Longitudinal study | Denmark | no | Adults | Built environment |
| Papagavriel et al., 2020 | Cross-sectional study | United Kingdom | yes | Adults | Social networks |
| Putrik et al., 2015 | Cross-sectional study | Netherlands | no | Adults | Access to service  Built environment  Mobility  Pollution Social networks |
| Ram et al., 2017 | Cross-sectional study | United Kingdom | no | Adults | Access to service Built environment  Security |
| Rask et al., 2018 | Cross-sectional study | Finland | no | Adults | Attitudinal factors towards vulnerable groups |
| Recio et al., 2021 | Cross-sectional study | Spain | yes | Adults | Attitudinal factors towards vulnerable groups |
| Reinhard et al., 2018 | Longitudinal study | United Kingdom | no | Adults | Mobility Policy |
| Riglin et al., 2019 | Longitudinal study | Ukraine | no | Children | Security |
| Rimes et al., 2019 | Cross-sectional study | United Kingdom | yes | Adolescents | Security  Social networks |
| Roberts 2014 | Cross-sectional study | Georgia | no | Adults | Policy |
| Roberts et al., 2019 | Longitudinal study | United Kingdom | no | youth <19 years | Pollution |
| Roberts et al., 2021 | Cross-sectional study | Netherlands | no | Adults | Built environment Security Social networks |
| Roccato & Russo, 2017 | Cross-sectional study | Italy | no | Adults | Security |
| Rocha et al., 2012 | Cross-sectional study | Spain | no | Adults | Built environment |
| Romani et al., 2021 | Cross-sectional study | Italy | yes | Adults | Attitudinal factors towards vulnerable groups |
| Rothon et al., 2012 | Cross-sectional study | United Kingdom | no | Adolescents | Social networks |
| Ruijsbroek et al., 2017 | Cross-sectional study | United Kingdom Spain Lithuania Netherlands | no | Adults | Built environment  Deprivation Social networks |
| Sáez et al., 2019 | Cross-sectional study | Spain | no | Adults | Attitudinal factors towards vulnerable groups |
| Sangster Jokić & Bartolac, 2018 | Qualitative study | Croatia | yes | Adults | Attitudinal factors towards vulnerable groups |
| Sariaslan et al., 2015 | Longitudinal study | Sweden | no | Adults | Deprivation  Population structures |
| Sattler et al., 2020 | Cross-sectional study | Austria | no | Adults | Mobility |
| Sattler & Zeyen, 2021 | Cross-sectional study | Germany | yes | Adults | Attitudinal factors towards vulnerable groups |
| Saville, 2021 | Longitudinal study | United Kingdom | no | Adults | Deprivation  Social networks |
| Scandurra et al., 2017 | Cross-sectional study | Italy | yes | Adults | Attitudinal factors towards vulnerable groups Social networks |
| Schnittker, 2020 | Cross-sectional study | Spain | no | Adults | Social networks |
| Schrier et al., 2014 | Cross-sectional study | Netherlands | no | Adults | Population structures |
| Schubert et al., 2019 | Systematic Review | Europe | no | Children | Pollution |
| Schunck et al., 2015 | Cross-sectional study | Germany | yes | Adults | Attitudinal factors towards vulnerable groups |
| Sevillano et al., 2014 | Cross-sectional study | Spain | yes | Adults | Attitudinal factors towards vulnerable groups Deprivation |
| Signoretta et al., 2019 | Cross-sectional study | Europe | no | Adults | Pollution |
| Simons et al., 2020 | Longitudinal study | Netherlands | no | Adults | Social networks |
| Singh et al., 2015 | Qualitative study | Georgia | yes | Adults | Attitudinal factors towards vulnerable groups Social networks |
| Slotman et al., 2017 | Cross-sectional study | Netherlands | yes | Adults | Attitudinal factors towards vulnerable groups |
| Smith et al., 2018 | Mixed methods | United Kingdom | yes | Adults | Social networks |
| Smith et al., 2020 | Cross-sectional study | United Kingdom | yes | Adults | Social networks |
| Sørensen et al., 2013 | Systematic review | Norway | no | Adults | Social networks |
| Spahlholz et al., 2016 | Cross-sectional study | Germany | yes | Adults | Attitudinal factors towards vulnerable groups |
| Stathopoulou et al., 2018 | Cross-sectional study | Greece | yes | Adults | Attitudinal factors towards vulnerable groups Macroeconomic environment Social networks |
| Steel et al., 2017 | Cross-sectional study | Sweden | yes | Adults | Attitudinal factors towards vulnerable groups Social networks |
| Stepanikova & Kukla, 2017 | Longitudinal study | Czechia | yes | Adults | Attitudinal factors towards vulnerable groups |
| Stojanovski et al., 2018 | Cross-sectional study | Macedonia | yes | Adults | Attitudinal factors towards vulnerable groups |
| Stronks et al., 2020 | Longitudinal study | Netherlands | yes | Adults | Attitudinal factors towards vulnerable groups Policy |
| Swanson et al., 2016 | Cross-sectional study | United Kingdom | no | Adults | Built environment |
| Tan & Haining, 2016 | Cross-sectional study | United Kingdom | no | Adults | Security |
| Taylor et al., 2013 | Cross-sectional study | Ireland | yes | Adults | Security |
| Thomas, 2015 | Qualitative study | Denmark | no | Adults | Built environment |
| Thoresen et al., 2018 | Cross-sectional study | Norway | yes | Adults | Social networks |
| Timmins et al., 2018 | Cross-sectional study | United Kingdom | yes | Adults | Attitudinal factors towards vulnerable groups |
| Van den Berg et al., 2016 | Cross-sectional study | Spain Netherlands Lithuania United Kingdom | no | Adults | Built environment |
| Van den Berg et al., 2019 | Cross-sectional study | Spain Netherlands Lithuania United Kingdom | no | Adults | Built environment |
| Verelst et al., 2022 | Longitudinal study | Finland Sweden United Kingdom | yes | Adolescents | Attitudinal factors towards vulnerable groups Social networks |
| Vilhjalmsdottir et al., 2016 | Cross-sectional study | Iceland | no | Adolescents | Social networks |
| Voci et al., 2017 | Cross-sectional study | Bosnia and Herzegovina | yes | Adults | Security |
| Weimann et al., 2015 | Longitudinal study | Sweden | no | Adults | Built environment |
| White et al., 2013 | Cross-sectional study | United Kingdom | no | Adults | Built environment |
| Wickham et al., 2014 | Cross-sectional study | United Kingdom | no | Adults | Deprivation Social networks |
| Wiginton et al., 2021 | Cross-sectional study | United Kingdom | yes | Adults | Access to service  Attitudinal factors towards vulnerable groups |
| Wind & Komproe, 2012 | Cross-sectional study | United Kingdom | yes | Adults | Climate change  Social networks |
| Wothge & Niemann, 2020 | Review | Germany | no | Adults | Pollution |
| Wu et al., 2017 | Cross-sectional study | United Kingdom | no | Adults | Built environment |
| Wypych & Bilewicz, 2022 | Longitudinal study | Poland | yes | Adults | Attitudinal factors towards vulnerable groups |
| Ye et al., 2021 | Longitudinal study | United Kingdom | no | Adults | Deprivation |
| Zhou et al., 2019 | Cross-sectional study | Macedonia | yes | Adults | Attitudinal factors towards vulnerable groups |

**References**

Abebe, D. S., Lien, L., & Hjelde, K. H. (2014). What we know and don’t know about mental health problems among immigrants in Norway. *Journal of Immigrant and Minority Health*, *16*(1), 60–67. https://pubmed.ncbi.nlm.nih.gov/23117694/

Adriaanse, M., Doreleijers, T., Domburgh, L., & Veling, W. (2016). Factors associated with psychiatric symptoms and psychiatric disorders in ethnic minority youth. *European Child & Adolescent Psychiatry*, *25*(10), 1067–1079. %22m.adriaanse@parnassiagroep.nl%22]

Adriaanse, M., Veling, W., Doreleijers, T., & van Domburgh, L. (2014). The link between ethnicity, social disadvantage and mental health problems in a school-based multiethnic sample of children in The Netherlands. *European Child & Adolescent Psychiatry*, *23*(11), 1103–1113. https://doi.org/10.1007/S00787-014-0564-5

Ahnquist, J., Wamala, S. P., & Lindstrom, M. (2012). Social determinants of health – A question of social or economic capital? Interaction effects of socioeconomic factors on health outcomes. *Social Science & Medicine*, *74*(6), 930–939. %22johanna.ahnquist@fhi.se%22]

Aichberger, M. C., Bromand, Z., Rapp, M. A., Yesil, R., Montesinos, A. H., Temur-Erman, S., Heinz, A., & Schouler-Ocak, M. (2015). Perceived ethnic discrimination, acculturation, and psychological distress in women of Turkish origin in Germany. *Social Psychiatry and Psychiatric Epidemiology: The International Journal for Research in Social and Genetic Epidemiology and Mental Health Services*, *50*(11), 1691–1700. %22marion.aichberger@charite.de%22]

Almquist, Y. B., Östberg, V., Rostila, M., Edling, C., & Rydgren, J. (2014). Friendship network characteristics and psychological well-being in late adolescence: Exploring differences by gender and gender composition. *Scandinavian Journal of Public Health*, *42*(2), 146–154. 0000-0002-3909-1080%22,

Astell-Burt, T., Mitchell, R., & Hartig, T. (2014). The association between green space and mental health varies across the lifecourse A longitudinal study. *Journal of Epidemiology and Community Health*, *68*(6), 578–583. %22T.Astell-Burt@uws.edu.au%22]

Bailey, J., Taylor, L., Kingston, P., & Watts, G. (2021). Older adults and “scams”: Evidence from the mass observation archive. *Journal of Adult Protection*, *23*(1), 57–69. %22g.watts@chester.ac.uk%22,

Bakic, H., & Ajdukovic, D. (2019). Stability and change post-disaster: Dynamic relations between individual, interpersonal and community resources and psychosocial functioning. *European Journal of Psychotraumatology*, *10*(1). %22hbakic@ffzg.hr%22]

Bakic, H., & Ajdukovic, D. (2021). Resilience after natural disasters: The process of harnessing resources in communities differentially exposed to a flood. *European Journal of Psychotraumatology*, *12*(1). %22hbakic@ffzg.hr%22]

Bamford, J., Klabbers, G., Curran, E., Rosato, M., & Leavey, G. (2021). Social capital and mental health among Black and minority ethnic groups in the UK. *Journal of Immigrant and Minority Health*, *23*(3), 502–510. 0000-0003-4897-2039%22,

Baranyi, G., Sieber, S., Pearce, J., Cheval, B., Dibben, C., Kliegel, M., & Cullati, S. (2019). A longitudinal study of neighbourhood conditions and depression in ageing European adults: Do the associations vary by exposure to childhood stressors? *Preventive Medicine: An International Journal Devoted to Practice and Theory*, *126*. 0000-0002-3881-446X%22,

Barratt, C., Green, G., & Speed, E. (2015). Mental health and houses in multiple occupation. *Journal of Public Mental Health*, *14*(2), 107–117. 0000-0002-3850-922X%22,

Barriuso-Lapresa, L., Hernando-Arizaleta, L., & Rajmil, L. (2012). Social inequalities in mental health and health-related quality of life in children in Spain. *Pediatrics*, *130*(3), e528–e535. 0000-0002-6625-0649%22,

Bastos, A. M., Faria, C., Moreira, E., Morais, D., Melo-de-Carvalho, J. M., & Paul, M. C. (2015). The importance of neighborhood ecological assets in community dwelling old people aging outcomes: A study in Northern Portugal. *Frontiers in Aging Neuroscience*, *7*(JUL), 156. https://doi.org/10.3389/FNAGI.2015.00156/BIBTEX

Borsch, A. S., de Montgomery, C. J., Gauffin, K., Eide, K., Heikkilä, E., & Smith Jervelund, S. (2019). Health, Education and Employment Outcomes in Young Refugees in the Nordic Countries: A Systematic Review. *Scandinavian Journal of Public Health*, *47*(7), 735–747. https://doi.org/10.1177/1403494818787099

Botchkovar, E. V, Antonaccio, O., & Hughes, L. A. (2018). Neighbourhood disorder, collective sentiments and personal strain: Bringing neighbourhood context into general strain theory. *British Journal of Criminology*, *58*(2), 455–477. https://search.ebscohost.com/login.aspx?direct=true&db=psyh&AN=2018-63607-011&site=ehost-live

Castaneda, A. E., Rask, S., Koponen, P., Suvisaari, J., Koskinen, S., Härkänen, T., Mannila, S., Laitinen, K., Jukarainen, P., & Jasinskaja-Lahti, I. (2015). The association between discrimination and psychological and social well-being: A population-based study of Russian, Somali and Kurdish migrants in Finland. *Psychology and Developing Societies*, *27*(2), 270–292. %22anu.castaneda@thl.fi%22]

Cecil, C. A. M., Viding, E., Barker, E. D., Guiney, J., & McCrory, E. J. (2014). Double disadvantage: The influence of childhood maltreatment and community violence exposure on adolescent mental health. *Journal of Child Psychology and Psychiatry*, *55*(7), 839–848. 0000-0002-2389-5922%22,

Chaves, C., Castellanos, T., Abrams, M., & Vazquez, C. (2018). The impact of economic recessions on depression and individual and social well-being: The case of Spain (2006–2013). *Social Psychiatry and Psychiatric Epidemiology: The International Journal for Research in Social and Genetic Epidemiology and Mental Health Services*, *53*(9), 977–986. 0000-0003-1796-2161%22,

Cramer, S., Losert, C., Schmau, M., & Kilian, R. (2013). Associations between community characteristics and psychiatric admissions in an urban area. *Social Psychiatry and Psychiatric Epidemiology*, *48*(11), 1797–1808. https://doi.org/10.1007/S00127-013-0667-1

Cramm, J. M., van Dijk, H. M., & Nieboer, A. P. (2013). Het belang van sociale cohesie en sociaal kapitaal in de buurt voor het welzijn van ouderen = The importance of neighborhood social cohesion and social capital for the well being of older adults in the community. *Tijdschrift Voor Gerontologie En Geriatrie*, *44*(2), 50–58. %22nieboer@bmg.eur.nl%22]

Curtis, S., Pain, R., Fuller, S., Khatib, Y., Rothon, C., Stansfeld, S. A., & Daya, S. (2013). Neighbourhood risk factors for Common Mental Disorders among young people aged 10-20 years: a structured review of quantitative research. *Health & Place*, *20*, 81–90. https://pubmed.ncbi.nlm.nih.gov/23399851/

Dabbagh, N., Johnson, S., King, M., & Blizard, R. (2012). Muslim adolescent mental health in the UK: An exploratory cross-sectional school survey. *International Journal of Culture and Mental Health*, *5*(3), 202–218. %22ndabbagh@hotmail.com%22]

de Freitas, Daniela Fonseca, Fernandes-Jesus, M., Ferreira, P. D., Coimbra, S., Teixeira, P. M., de Moura, A., Gato, J., Marques, S. C., & Fontaine, A. M. (2018). Psychological correlates of perceived ethnic discrimination in Europe: A meta-analysis. *Psychology of Violence*, *8*(6), 712–725. 0000-0002-8876-4595%22,

de Vries, S., van Dillen, S. M. E., Groenewegen, P. P., & Spreeuwenberg, P. (2013). Streetscape greenery and health: Stress, social cohesion and physical activity as mediators. *Social Science & Medicine*, *94*, 26–33. 0000-0003-2127-8442%22,

Delaruelle, K., Walsh, S. D., Dierckens, M., Deforche, B., Kern, M. R., Currie, C., Maldonado, C. M., Cosma, A., & Stevens, G. W. J. M. (2021). Mental health in adolescents with a migration background in 29 European countries: The buffering role of social capital. *Journal of Youth and Adolescence*, *50*(5), 855–871. 0000-0001-6726-6952%22,

Derdikman‐Eiron, R., Hjemdal, O., Lydersen, S., Bratberg, G. H., & Indredavik, M. S. (2013). Adolescent predictors and associates of psychosocial functioning in young men and women: 11 year follow‐up findings from the Nord‐Trøndelag Health Study. *Scandinavian Journal of Psychology*, *54*(2), 95–101. 0000-0002-6430-2345%22,

Dewaele, A., Van Houtte, M., & Vincke, J. (2014). Visibility and coping with minority stress: A gender-specific analysis among lesbians, gay men, and bisexuals in Flanders. *Archives of Sexual Behavior*, *43*(8), 1601–1614. %22alexis.dewaele@ugent.be%22]

Domènech-Abella, J., Mundó, J., Leonardi, M., Chatterji, S., Tobiasz-Adamczyk, B., Koskinen, S., Ayuso-Mateos, J. L., Haro, J. M., & Olaya, B. (2020). Loneliness and depression among older European adults: The role of perceived neighborhood built environment. *Health & Place*, *62*. https://doi.org/10.1016/J.HEALTHPLACE.2019.102280

Donisi, V., Tedeschi, F., Percudani, M., Fiorillo, A., Confalonieri, L., De Rosa, C., Salazzari, D., Tansella, M., Thornicroft, G., & Amaddeo, F. (2013). Prediction of community mental health service utilization by individual and ecological level socio-economic factors. *Psychiatry Research*, *209*(3), 691–698. https://doi.org/10.1016/J.PSYCHRES.2013.02.031

Economou, M., Madianos, M., Peppou, L. E., Souliotis, K., Patelakis, A., & Stefanis, C. (2014). Cognitive social capital and mental illness during economic crisis: A nationwide population-based study in Greece. *Social Science & Medicine*, *100*, 141–147. %22lilly.peppou@gmail.com%22,

Economou, M., Souliotis, K., Peppou, L. E., Agapidaki, I., Tzavara, C., & Stefanis, C. N. (2018). Major depression in Cyprus amid financial crisis: Prevalence and correlates. *International Journal of Culture and Mental Health*, *11*(3), 255–267. %22antistigma@epipsi.eu%22]

Elliott, J., Gale, C. R., Parsons, S., & Kuh, D. (2014). Neighbourhood cohesion and mental wellbeing among older adults: a mixed methods approach. *Social Science & Medicine (1982)*, *107*, 44–51. https://doi.org/10.1016/J.SOCSCIMED.2014.02.027

Engemann, K., Pedersen, C. B., Agerbo, E., Arge, L., Børglum, A. D., Erikstrup, C., Hertel, O., Hougaard, D. M., McGrath, J. J., Mors, O., Mortensen, P. B., Nordentoft, M., Sabel, C. E., Sigsgaard, T., Tsirogiannis, C., Vilhjálmsson, B. J., Werge, T., Svenning, J.-C., & Horsdal, H. T. (2020). Association between childhood green space, genetic liability, and the incidence of schizophrenia. *Schizophrenia Bulletin*, *46*(6), 1629–1637. 0000-0003-1519-8091%22,

Engemann, K., Pedersen, C. B., Arge, L., Tsirogiannis, C., Mortensen, P. B., & Svenning, J.-C. (2019). Residential green space in childhood is associated with lower risk of psychiatric disorders from adolescence into adulthood. *PNAS Proceedings of the National Academy of Sciences of the United States of America*, *116*(11), 5188–5193. 0000-0003-1431-1726%22,

Essers, E., Pérez-Crespo, L., Foraster, M., Ambrós, A., Tiemeier, H., & Guxens, M. (2022). Environmental noise exposure and emotional, aggressive, and attention-deficit/hyperactivity disorder-related symptoms in children from two European birth cohorts. *Environment International*, *158*. https://doi.org/10.1016/J.ENVINT.2021.106946

Ettema, D., & Schekkerman, M. (2016). How do spatial characteristics influence well-being and mental health? Comparing the effect of objective and subjective characteristics at different spatial scales. *Travel Behaviour and Society*, *5*, 56–67. %22d.f.ettema@uu.nl%22]

Finegan, M., Firth, N., & Delgadillo, J. (2020). Adverse impact of neighbourhood socioeconomic deprivation on psychological treatment outcomes: The role of area-level income and crime. *Psychotherapy Research*, *30*(4), 546–554. 0000-0001-5349-230X%22,

Fone, D., White, J., Farewell, D., Kelly, M., John, G., Lloyd, K., Williams, G., & Dunstan, F. (2014). Effect of neighborhood deprivation and social cohesion on mental health inequality: A multilevel population-based longitudinal study. *Psychological Medicine*, *44*(11), 2449–2460. 0000-0002-1440-4124%22,

Forsman, A. K., Nyqvist, F., Schierenbeck, I., Gustafson, Y., & Wahlbeck, K. (2012). Structural and cognitive social capital and depression among older adults in two nordic regions. *Aging & Mental Health*, *16*(6), 771–779. 0000-0002-0999-377X%22,

Foy, A. A. J., Morris, D., Fernandes, V., & Rimes, K. A. (2019). LGBQ+ adults’ experiences of improving access to psychological therapies and primary care counselling services: Informing clinical practice and service delivery. *The Cognitive Behaviour Therapist*, *12*. 0000-0003-2634-455X%22,

Freitas, Daniela F, D’Augelli, A. R., Coimbra, S., & Fontaine, A. M. (2016). Discrimination and mental health among gay, lesbian, and bisexual youths in Portugal: The moderating role of family relationships and optimism. *Journal of GLBT Family Studies*, *12*(1), 68–90. 0000-0002-7738-4757%22,

Friborg, O., Sørlie, T., & Hansen, K. L. (2017). Resilience to discrimination among indigenous Sami and non-Sami populations in Norway: The SAMINOR2 study. *Journal of Cross-Cultural Psychology*, *48*(7), 1009–1027. 0000-0001-6629-0782%22,

Generaal, E., Hoogendijk, E. O., Stam, M., Henke, C. E., Rutters, F., Oosterman, M., Huisman, M., Kramer, S. E., Elders, P. J. M., Timmermans, E. J., Lakerveld, J., Koomen, E., ten Have, M., de Graaf, R., Snijder, M. B., Stronks, K., Willemsen, G., Boomsma, D. I., Smit, J. H., & Penninx, B. W. J. H. (2019). Neighbourhood characteristics and prevalence and severity of depression: Pooled analysis of eight Dutch cohort studies. *The British Journal of Psychiatry*, *215*(2), 468–475. %22b.penninx@vumc.nl%22]

Generaal, E., Timmermans, E. J., Dekkers, J. E. C., Smit, J. H., & Penninx, B. W. J. H. (2019). Not urbanization level but socioeconomic, physical and social neighbourhood characteristics are associated with presence and severity of depressive and anxiety disorders. *Psychological Medicine*, *49*(1), 149–161. %22e.generaal@ggzingeest.nl%22]

Gnan, G. H., Rahman, Q., Ussher, G., Baker, D., West, E., & Rimes, K. A. (2019). General and LGBTQ-specific factors associated with mental health and suicide risk among LGBTQ students. *Journal of Youth Studies*, *22*(10), 1393–1408. 0000-0003-2634-455X%22,

Gubbels, J. S., Kremers, S. P. J., Droomers, M., Hoefnagels, C., Stronks, K., Hosman, C., & de Vries, S. (2016). The impact of greenery on physical activity and mental health of adolescent and adult residents of deprived neighborhoods: A longitudinal study. *Health & Place*, *40*, 153–160. %22s.kremers@maastrichtuniversity.nl%22,

Gube, M., Esser, A., Schettgen, T., Quinete, N., Bertram, J., Putschogl, F. M., Kraus, T., & Lang, J. (2019). Depressive symptoms after PCB exposure: Hypotheses for underlying pathomechanisms via the thyroid and dopamine system. *International Journal of Environmental Research and Public Health*, *16*(6), 950. https://www.mdpi.com/1660-4601/16/6/950/pdf

Hackett, R. A., Steptoe, A., & Jackson, S. E. (2019). Sex discrimination and mental health in women: A prospective analysis. *Health Psychology*, *38*(11), 1014–1024. 0000-0002-5428-2950%22,

Hamano, T., Li, X., Lönn, S. L., Nabika, T., Sundquist, J., & Sundquist, K. (2018). Is familial risk for depression confounded by individual and familial socioeconomic factors and neighborhood environmental factors? A 7-year follow-up study in Sweden. *Psychiatry Research*, *266*, 30. https://doi.org/10.1016/J.PSYCHRES.2018.05.041

Hansen, K. L., & Sørlie, T. (2012). Ethnic discrimination and psychological distress: A study of Sami and non-Sami populations in Norway. *Transcultural Psychiatry*, *49*(1), 26–50. %22ketil.lenert.hansen@uit.no%22]

Hatch, S. L., Gazard, B., Williams, D. R., Frissa, S., Goodwin, L., & Hotopf, M. (2016). Discrimination and common mental disorder among migrant and ethnic groups: Findings from a South East London Community sample. *Social Psychiatry and Psychiatric Epidemiology: The International Journal for Research in Social and Genetic Epidemiology and Mental Health Services*, *51*(5), 689–701. 0000-0002-3980-4466%22,

Helsen, V., Enzlin, P., & Gijs, L. (2021). Mental health in transgender adults: The role of proximal minority stress, community connectedness, and gender nonconformity. *Psychology of Sexual Orientation and Gender Diversity*. 0000-0002-6641-5159%22,

Hüfner, K., Ower, C., Kemmler, G., Vill, T., Martini, C., Schmitt, A., & Sperner-Unterweger, B. (2020). Viewing an alpine environment positively affects emotional analytics in patients with somatoform, depressive and anxiety disorders as well as in healthy controls. *BMC Psychiatry*, *20*. 0000-0002-5453-8792%22,

Igde, E., Heinz, A., Schouler-Ocak, M., & Rössler, W. (2019). Depressive und somatoforme Störungen bei türkeistämmigen Personen in Deutschland = Depressive and somatoform disorders in persons with a Turkish migration background in Germany. *Der Nervenarzt*, *90*(1), 25–34. %22ela.igde@charite.de%22]

Ikram, U. Z., Snijder, M. B., Fassaert, T. J. L., Schene, A. H., Kunst, A. E., & Stronks, K. (2015). The contribution of perceived ethnic discrimination to the prevalence of depression. *European Journal of Public Health*, *25*(2), 243–248. %22u.ikram@amc.uva.nl%22]

Infurna, M. R., Giannone, F., Guarnaccia, C., Lo Cascio, M., Parzer, P., & Kaess, M. (2015). Environmental factors that distinguish between clinical and healthy samples with childhood experiences of abuse and neglect. *Psychopathology*, *48*(4), 256–263. %22michael.kaess@med.uni-heidelberg.de%22]

J.M., M., J.R.I., C., G., B., & E., V. (2021). Association between Genetic Risk for Psychiatric Disorders and the Probability of Living in Urban Settings. *JAMA Psychiatry*, *78*(12), 1355–1364. http://archpsyc.jamanetwork.com/issues.aspx

Jackson, S. E., Hackett, R. A., Grabovac, I., Smith, L., & Steptoe, A. (2019). Perceived discrimination, health and wellbeing among middle-aged and older lesbian, gay and bisexual people: A prospective study. *PLoS ONE*, *14*(5), e0216497. https://journals.plos.org/plosone/article/file?id=10.1371/journal.pone.0216497&type=printable

Jonsson, K. R., Vartanova, I., & Södergren, M. (2018). Ethnic variations in mental health among 10-15-year-olds living in England and Wales: The impact of neighbourhood characteristics and parental behaviour. *Health & Place*, *51*, 189–199. https://doi.org/10.1016/J.HEALTHPLACE.2018.03.010

Junghans, C., & Jones, M. (2007). Consent bias in research: how to avoid it. *Heart (British Cardiac Society)*, *93*(9), 1024–1025. https://doi.org/10.1136/hrt.2007.120113

Källström, Å., Hellfeldt, K., Howell, K. H., Miller-Graff, L. E., & Graham-Bermann, S. A. (2020). Young adults victimized as children or adolescents: Relationships between perpetrator patterns, poly-victimization, and mental health problems. *Journal of Interpersonal Violence*, *35*(11), 2335–2357. %22asa.kallstrom@oru.se%22]

Kendrick, K., Jutengren, G., & Stattin, H. (2012). The protective role of supportive friends against bullying perpetration and victimization. *Journal of Adolescence*, *35*(4), 1069–1080. %22hakan.stattin@oru.se%22,

Klaperski, S., Koch, E., Hewel, D., Schempp, A., & Müller, J. (2019). Optimizing mental health benefits of exercise: The influence of the exercise environment on acute stress levels and wellbeing. *Mental Health and Prevention*, *15*. 0000-0002-1718-1392%22,

Knott, C. S., Panter, J., Foley, L., & Ogilvie, D. (2018). Changes in the mode of travel to work and the severity of depressive symptoms: A longitudinal analysis of UK Biobank. *Preventive Medicine: An International Journal Devoted to Practice and Theory*, *112*, 61–69. 0000-0003-3028-7340%22,

Kreski, N. T., Park, S. H., Safren, S. A., Goedel, W. C., Morganstein, J. G., Chaix, B., & Duncan, D. T. (2018). Is neighborhood safety associated with depression symptoms, anxiety symptoms, and psychological distress among gay, bisexual, and other men who have sex with men? *Journal of Gay & Lesbian Mental Health*, *22*(3), 243–260. 0000-0002-2869-1048%22,

Kritsotakis, G., Vassilaki, M., Melaki, V., Georgiou, V., Philalithis, A. E., Bitsios, P., Kogevinas, M., Chatzi, L., & Koutis, A. (2013). Social capital in pregnancy and postpartum depressive symptoms: A prospective mother–child cohort study (the Rhea study). *International Journal of Nursing Studies*, *50*(1), 63–72. 0000-0002-3711-3936%22,

Kunst, M., & Van Wilsem, J. (2013). Trait impulsivity and change in mental health problems after violent crime victimization: A prospective analysis of the Dutch longitudinal internet studies for the social sciences database. *Journal of Interpersonal Violence*, *28*(8), 1642–1656. %22m.j.j.kunst@law.leidenuniv.nl%22]

Lafferty, A., Duryae, E.L., Martin, R., Moseley, L., Wafford, M., McIntire, D.D., Sponge, C.Y., & Nelson, D.B. (2022). A Prospective Study of Social Needs Associated with Mental Health Among Mothers Living in Poverty. American Journal of Obstetrics and Gynecology, 226(1), S593–S594.

Lakeman, R., McGowan, P., MacGabhann, L., Parkinson, M., Redmond, M., Sibitz, I., Stevenson, C., & Walsh, J. (2012). A qualitative study exploring experiences of discrimination associated with mental-health problems in Ireland. *Epidemiology and Psychiatric Sciences*, *21*(3), 271–279. %22Richard.lakeman@scu.edu.au%22]

Landstedt, E., Almquist, Y. B., Eriksson, M., & Hammarström, A. (2016). Disentangling the directions of associations between structural social capital and mental health: Longitudinal analyses of gender, civic engagement and depressive symptoms. *Social Science & Medicine*, *163*, 135–143. %22evelina.landstedt@umu.se%22]

Lanfredi, M., Zoppei, S., Ferrari, C., Bonetto, C., Van Bortel, T., Thornicroft, G., Knifton, L., Quinn, N., Rossi, G., & Lasalvia, A. (2015). Self-stigma as a mediator between social capital and empowerment among people with major depressive disorder in Europe: The ASPEN study. *European Psychiatry*, *30*(1), 58–64. 0000-0002-5902-2568%22,

Latham, R. M., Kieling, C., Arseneault, L., Botter-Maio Rocha, T., Beddows, A., Beevers, S. D., Danese, A., De Oliveira, K., Kohrt, B. A., Moffitt, T. E., Mondelli, V., Newbury, J. B., Reuben, A., & Fisher, H. L. (2021). Childhood exposure to ambient air pollution and predicting individual risk of depression onset in UK adolescents. *Journal of Psychiatric Research*, *138*, 60–67. 0000-0003-4174-2126%22,

Lecerof, S. S., Stafström, M., Westerling, R., & Östergren, P.-O. (2016). Does social capital protect mental health among migrants in Sweden? *Health Promotion International*, *31*(3), 644–652. %22susanne.sundell_lecerof@med.lu.se%22]

Lee, E. J., Keyes, K., Bitfoi, A., Mihova, Z., Pez, O., Yoon, E., & Masfety, V. K. (2014). Mental health disparities between Roma and non-Roma children in Romania and Bulgaria. *BMC Psychiatry*, *14*. %22viviane.kovess@ehesp.fr%22,

Levecque, K., & Van Rossem, R. (2015). Depression in Europe: Does migrant integration have mental health payoffs? A cross-national comparison of 20 European countries. *Ethnicity & Health*, *20*(1), 49–65. %22Katia.Levecque@UGent.be%22]

Lindström, M., & Giordano, G. N. (2016). The 2008 financial crisis: Changes in social capital and its association with psychological wellbeing in the United Kingdom—A panel study. *Social Science & Medicine*, *153*, 71–80. %22Giuseppe_nicola.giordano@med.lu.se%22,

Lindström, M., & Rosvall, M. (2012). Marital status, social capital, economic stress, and mental health: A population-based study. *The Social Science Journal*, *49*(3), 339–342. %22martin.lindstrom@med.lu.se%22]

Llorente, J. M., Oliván-Blázquez, B., Zuñiga-Antón, M., Masluk, B., Andrés, E., García-Campayo, J., & Magallón-Botaya, R. (2018). Variability of the prevalence of depression in function of sociodemographic and environmental factors: Ecological model. *Frontiers in Psychology*, *9*. %22bolivan@unizar.es%22]

Magallares, A., Bolaños-Rios, P., Ruiz-Prieto, I., de Valle, P., Irles, J. A., & Jáuregui-Lobera, I. (2017). The mediational effect of weight self-stigma in the relationship between blatant and subtle discrimination and depression and anxiety. *The Spanish Journal of Psychology*, *20*. %22amagallares@psi.uned.es%22]

Magallares, A., Morales, J. F., & Rubio, M. Á. (2014). Group identification, discrimination and psychological health in an obese sample. *International Journal of Psychology & Psychological Therapy*, *14*(3), 421–431. %22amagallares@psi.uned.es%22]

Mangalore, R., & Knapp, M. (2012). Income-related inequalities in common mental disorders among ethnic minorities in England. *Social Psychiatry and Psychiatric Epidemiology: The International Journal for Research in Social and Genetic Epidemiology and Mental Health Services*, *47*(3), 351–359. %22R.K.Mangalore@lse.ac.uk%22]

Markkula, N., Härkänen, T., Nieminen, T., Peña, S., Mattila, A. K., Koskinen, S., Saarni, S. I., & Suvisaari, J. (2016). Prognosis of depressive disorders in the general population– results from the longitudinal Finnish Health 2011 Study. *Journal of Affective Disorders*, *190*, 687–696. 0000-0001-7167-0990%22,

McEachan, R. R. C., Prady, S. L., Smith, G., Fairley, L., Cabieses, B., Gidlow, C., Wright, J., Dadvand, P., van Gent, D., & Nieuwenhuijsen, M. J. (2016). The association between green space and depressive symptoms in pregnant women: Moderating roles of socioeconomic status and physical activity. *Journal of Epidemiology and Community Health*, *70*(3), 253–259. 0000-0001-9572-7293%22,

McGorrian, C., Hamid, N. A., Fitzpatrick, P., Daly, L., Malone, K. M., & Kelleher, C. (2013). Frequent mental distress (FMD) in Irish travellers: Discrimination and bereavement negatively influence mental health in the all Ireland traveller health study. *Transcultural Psychiatry*, *50*(4), 559–578. 0000-0003-2524-3677%22,

Mears, M., Brindley, P., Jorgensen, A., & Maheswaran, R. (2020). Population-level linkages between urban greenspace and health inequality: The case for using multiple indicators of neighbourhood greenspace. *Health & Place*, *62*. 0000-0002-3899-4421%22,

Mewes, R., Asbrock, F., & Laskawi, J. (2015). Perceived discrimination and impaired mental health in Turkish immigrants and their descendents in Germany. *Comprehensive Psychiatry*, *62*, 42–50. %22mewesr@uni-marburg.de%22]

Missinne, S., & Bracke, P. (2012). Depressive symptoms among immigrants and ethnic minorities: A population based study in 23 European countries. *Social Psychiatry and Psychiatric Epidemiology: The International Journal for Research in Social and Genetic Epidemiology and Mental Health Services*, *47*(1), 97–109. 0000-0002-9477-3236%22,

Mölsä, M., Kuittinen, S., Tiilikainen, M., Honkasalo, M.-L., & Punamäki, R.-L. (2017). Mental health among older refugees: The role of trauma, discrimination, and religiousness. *Aging & Mental Health*, *21*(8), 829–837. 0000-0001-5613-7637%22,

Motoc, I., Timmermans, E. J., Deeg, D., Penninx, B. W. J. H., & Huisman, M. (2019). Associations of neighbourhood sociodemographic characteristics with depressive and anxiety symptoms in older age: Results from a 5-wave study over 15 years. *Health & Place*, *59*. %22i.motoc@amsterdamumc.nl%22]

Mueller, M. A. E., & Flouri, E. (2020). Neighbourhood greenspace and children’s trajectories of self-regulation: Findings from the UK Millennium Cohort Study. *Journal of Environmental Psychology*, *71*. 0000-0001-6207-4847%22,

Mueller, M. A. E., Flouri, E., & Kokosi, T. (2019). The role of the physical environment in adolescent mental health. *Health & Place*, *58*. 0000-0003-1590-6764%22,

Müller, L. R. F., Gossmann, K., Schmid, R. F., Rosner, R., & Unterhitzenberger, J. (2021). A pilot study on ecological momentary assessment in asylum-seeking children and adolescents resettled to Germany: Investigating compliance, post-migration factors, and the relation between daily mood, sleep patterns, and mental health. *PLoS ONE*, *16*(2). 0000-0003-0608-0534%22,

Nesterko, Y., Seidel, N., Brähler, E., & Claesmer, H. (2014). Depression und Angst bei älteren russischstammigen Menschen mit jüdischem Hintergrund in Deutschland: Wie wirken sich Diskriminierung und Religiosität aus? = Depression and anxiety in elderly Jews from the former Soviet Union in Germany: The role of discrimination and religiosity. *Psychiatrische Praxis*, *41*(2), 76–81. %22yurly.nesterko@uni-leipzig.de%22]

Newbury, J., Arseneault, L., Caspi, A., Moffitt, T. E., Odgers, C. L., & Fisher, H. L. (2016). Why are children in urban neighborhoods at increased risk for psychotic symptoms? Findings from a UK longitudinal cohort study. *Schizophrenia Bulletin*, *42*(6), 1372–1383. 0000-0002-8589-6760%22,

Newbury, J. B., Stewart, R., Fisher, H. L., Beevers, S., Dajnak, D., Broadbent, M., Pritchard, M., Shiode, N., Heslin, M., Hammoud, R., Hotopf, M., Hatch, S. L., Mudway, I. S., & Bakolis, I. (2021). Association between air pollution exposure and mental health service use among individuals with first presentations of psychotic and mood disorders: Retrospective cohort study. *The British Journal of Psychiatry*, *219*(6), 678–685. 0000-0002-4800-1630%22,

Nilsen, L. G., Thoresen, S., Wentzel-Larsen, T., & Dyb, G. (2019). Trust after terror: Institutional trust among young terror survivors and their parents after the 22nd of July terrorist attack on Utøya Island, Norway. *Frontiers in Psychology*, *10*. %22l.g.nilsen@nkvts.no%22]

Nimmo‐Smith, V., Brugha, T. S., Kerr, M. P., McManus, S., & Rai, D. (2016). Discrimination, domestic violence, abuse, and other adverse life events in people with epilepsy: Population‐based study to assess the burden of these events and their contribution to psychopathology. *Epilepsia*, *57*(11), 1870–1878. 0000-0002-7239-3523%22,

Noordzij, J. M., Beenackers, M. A., Oude Groeniger, J., Timmermans, E., Chaix, B., Doiron, D., Huisman, M., Motoc, I., Ruiz, M., Wissa, R., Avendano, M., & Van Lenthe, F. J. (2021). Green spaces, subjective health and depressed affect in middle-aged and older adults: a cross-country comparison of four European cohorts. *Journal of Epidemiology and Community Health*, *75*(5), 470–476. https://doi.org/10.1136/JECH-2020-214257

Nyqvist, F., Forsman, A. K., & Cattan, M. (2013). A comparison of older workers’ and retired older people’s social capital and sense of mastery. *Scandinavian Journal of Public Health*, *41*(8), 792–798. %22fredrica.nyqvist@thl.fi%22]

Oppedal, B., & Idsoe, T. (2015). The role of social support in the acculturation and mental health of unaccompanied minor asylum seekers. *Scandinavian Journal of Psychology*, *56*(2), 203–211. %22brit.oppedal@fhi.no%22]

Østergaard, S. D., Waltoft, B. L., Mortensen, P. B., & Mors, O. (2013). Environmental and familial risk factors for psychotic and non-psychotic severe depression. *Journal of Affective Disorders*, *147*(1), 232–240. 0000-0002-5230-9865%22,

Papagavriel, K., Jones, R., Sheehan, R., Hassiotis, A., & Ali, A. (2020). The association between loneliness and common mental disorders in adults with borderline intellectual impairment. *Journal of Affective Disorders*, *277*, 954–961. %22afia.ali@ucl.ac.uk%22,

Putrik, P., de Vries, N. K., Mujakovic, S., van Amelsvoort, L., Kant, Ij., Kunst, A. E., van Oers, H., & Jansen, M. (2015). Living environment matters: Relationships between neighborhood characteristics and health of the residents in a Dutch municipality. *Journal of Community Health: The Publication for Health Promotion and Disease Prevention*, *40*(1), 47–56. 0000-0003-4063-2240%22,

Ram, B., Shankar, A., Nightingale, C. M., Giles-Corti, B., Ellaway, A., Cooper, A. R., Page, A., Cummins, S., Lewis, D., Whincup, P. H., Cook, D. G., Rudnicka, A. R., & Owen, C. G. (2017). Comparisons of depression, anxiety, well-being, and perceptions of the built environment amongst adults seeking social, intermediate and market-rent accommodation in the former London Olympic Athletes’ Village. *Health & Place*, *48*, 31–39. https://doi.org/10.1016/J.HEALTHPLACE.2017.09.001

Rask, S., Elo, I. T., Koskinen, S., Lilja, E., Koponen, P., & Castaneda, A. E. (2018). The association between discrimination and health: Findings on Russian, Somali and Kurdish origin populations in Finland. *European Journal of Public Health*, *28*(5), 898–903. %22shadia.rask@thl.fi%22]

Recio, P., Molero, F., Silván-Ferrero, P., & Nouvilas-Pallejà, E. (2021). Perceived discrimination and emotional distress among family caregivers of children with physical disabilities: The mediational role of affiliate stigma and self-efficacy. *American Journal of Orthopsychiatry*, *91*(3), 367–374. 0000-0001-9645-5197%22,

Reinhard, E., Courtin, E., van Lenthe, F. J., & Avendano, M. (2018). Public transport policy, social engagement and mental health in older age: A quasi-experimental evaluation of free bus passes in England. *Journal of Epidemiology and Community Health*, *72*(5), 361–368. 0000-0002-7295-2911%22,

Riglin, L., Hammerton, G., Heron, J., Collishaw, S., Arseneault, L., Thapar, A. K., Maughan, B., O’Donovan, M. C., & Thapar, A. (2019). Developmental contributions of schizophrenia risk alleles and childhood peer victimization to early-onset mental health trajectories. *The American Journal of Psychiatry*, *176*(1), 36–43. 0000-0002-3689-737X%22,

Rimes, K. A., Shivakumar, S., Ussher, G., Baker, D., Rahman, Q., & West, E. (2019). Psychosocial factors associated with suicide attempts, ideation, and future risk in lesbian, gay, and bisexual youth: The Youth Chances study. *Crisis: The Journal of Crisis Intervention and Suicide Prevention*, *40*(2), 83–92. 0000-0001-8346-4529%22,

Roberts, H., van Lissa, C., & Helbich, M. (2021). Perceived neighbourhood characteristics and depressive symptoms: Potential mediators and the moderating role of employment status. *Social Science & Medicine*, *268*. 0000-0003-0392-8915%22,

Roberts, S., Arseneault, L., Barratt, B., Beevers, S., Danese, A., Odgers, C. L., Moffitt, T. E., Reuben, A., Kelly, F. J., & Fisher, H. L. (2019). Exploration of NO₂ and PM25 air pollution and mental health problems using high-resolution data in London-based children from a UK longitudinal cohort study. *Psychiatry Research*, *272*, 8–17. 0000-0003-4174-2126%22,

Roccato, M., & Russo, S. (2017). Right‐wing authoritarianism, societal threat to safety, and psychological distress. *European Journal of Social Psychology*, *47*(5), 600–610. %22michele.roccato@unito.it%22]

Rocha, K., Pérez, K., Rodríguez-Sanz, M., Obiols, J. E., & Borrell, C. (2012). Perception of environmental problems and common mental disorders (CMD). *Social Psychiatry and Psychiatric Epidemiology*, *47*(10), 1675–1684. https://doi.org/10.1007/S00127-012-0474-0

Romani, A., Mazzoli, F., Ristori, J., Cocchetti, C., Cassioli, E., Castellini, G., Mosconi, M., Meriggiola, M. C., Gualdi, S., Giovanardi, G., Lingiardi, V., Vignozzi, L., Maggi, M., & Fisher, A. D. (2021). Psychological wellbeing and perceived social acceptance in gender diverse individuals. *Journal of Sexual Medicine*, *18*(11), 1933–1944. 0000-0003-3623-7096%22,

Rothon, C., Goodwin, L., & Stansfeld, S. (2012). Family social support, community “social capital” and adolescents’ mental health and educational outcomes: A longitudinal study in England. *Social Psychiatry and Psychiatric Epidemiology: The International Journal for Research in Social and Genetic Epidemiology and Mental Health Services*, *47*(5), 697–709. %22c.rothon@qmul.ac.uk%22]

Ruijsbroek, A., Mohnen, S. M., Droomers, M., Kruize, H., Gidlow, C., Gražulevičiene, R., Andrusaityte, S., Maas, J., Nieuwenhuijsen, M. J., Triguero-Mas, M., Masterson, D., Ellis, N., Kempen, E., Hardyns, W., Stronks, K., & Groenewegen, P. P. (2017). Neighbourhood green space, social environment and mental health: An examination in four European cities. *International Journal of Public Health*, *62*(6), 657–667. 0000-0002-1580-2693%22,

Sáez, G., Valor-Segura, I., & Expósito, F. (2019). Interpersonal sexual objectification experiences: Psychological and social well-being consequences for women. *Journal of Interpersonal Violence*, *34*(4), 741–762. %22gemmasaez@ugr.es%22]

Sangster Jokić, C., & Bartolac, A. (2018). Iskustvo stresa i mentalno zdravlje osoba s tjelesnim invaliditetom: Perspektiva manjinskog stresa = The stress experience and mental health among persons with physical disabilities: A minority stress perspective. *Socijalna Psihijatrija*, *46*(1), 26–57. %22ClaireAlexandra.SangsterJokic@zvu.hr%22]

Sariaslan, A., Larsson, H., D’Onofrio, B., Långström, N., Fazel, S., & Lichtenstein, P. (2015). Does population density and neighborhood deprivation predict schizophrenia? A nationwide Swedish family-based study of 24 million individuals. *Schizophrenia Bulletin*, *41*(2), 494–502. 0000-0003-3037-5287%22,

Sattler, F. A., & Zeyen, J. (2021). Intersecting identities, minority stress, and mental health problems in different sexual and ethnic groups. *Stigma and Health*, *6*(4), 457–466. 0000-0001-8974-445X%22,

Sattler, M. C., Färber, T., Traußnig, K., Köberl, G., Paier, C., Dietz, P., & van Poppel, M. N. M. (2020). Cross-sectional association between active commuting and perceived commuting stress in Austrian adults: Results from the HOTway study. *Mental Health and Physical Activity*, *19*. 0000-0003-4576-682X%22,

Saville, C. W. N. (2021). Ecological social capital does not predict geographical variance in increases in depression following the 2008 financial crisis. *British Journal of Psychology*, *112*(1), 163–179. 0000-0003-4870-7630%22,

Scandurra, C., Amodeo, A. L., Valerio, P., Bochicchio, V., & Frost, D. M. (2017). Minority stress, resilience, and mental health: A study of Italian transgender people. *Journal of Social Issues*, *73*(3), 563–585. 0000-0003-2788-1381%22,

Schnittker, J. (2020). Religion, social integration, and depression in Europe: Evidence from the European Social Survey. *Social Science & Medicine*, *267*. %22jschnitt@ssc.upenn.edu%22]

Schrier, A. C., Peen, J., de Wit, M. A. S., van Ameijden, E. J. C., Erdem, Ö., Verhoeff, A. P., Dekker, J. J. M., & Beekman, A. T. F. (2014). Ethnic density is not associated with psychological distress in Turkish-Dutch, Moroccan-Dutch and Surinamese-Dutch ethnic minorities in the Netherlands. *Social Psychiatry and Psychiatric Epidemiology: The International Journal for Research in Social and Genetic Epidemiology and Mental Health Services*, *49*(10), 1557–1567. %22A.Schrier@i-psy.nl%22]

Schubert, M., Hegewald, J., Freiberg, A., Starke, K. R., Augustin, F., Riedel-Heller, S. G., Zeeb, H., & Seidler, A. (2019). Behavioral and Emotional Disorders and Transportation Noise among Children and Adolescents: A Systematic Review and Meta-Analysis. *International Journal of Environmental Research and Public Health*, *16*(18). https://doi.org/10.3390/IJERPH16183336

Schunck, R., Reiss, K., & Razum, O. (2015). Pathways between perceived discrimination and health among immigrants: Evidence from a large national panel survey in Germany. *Ethnicity & Health*, *20*(5), 493–510. %22reinhard.schunck@uni-bielefeld.de%22]

Sevillano, V., Basabe, N., Bobowik, M., & Aierdi, X. (2014). Health-related quality of life, ethnicity and perceived discrimination among immigrants and natives in Spain. *Ethnicity & Health*, *19*(2), 178–197. 0000-0003-4753-4299%22,

Signoretta, P. E., Buffel, V., & Bracke, P. (2019). Mental wellbeing, air pollution and the ecological state. *Health & Place*, *57*, 82–91. %22p.e.signoretta@lboro.ac.uk%22]

Simons, M., Lataster, J., Reijnders, J., Peeters, S., Janssens, M., & Jacobs, N. (2020). Bonding personal social capital as an ingredient for positive aging and mental well-being A study among a sample of Dutch elderly. *Aging & Mental Health*, *24*(12), 2034–2042. 0000-0003-4021-4014%22,

Singh, G. K., Siahpush, M., Azuine, R. E., & Williams, S. D. (2015). Increasing Area Deprivation and Socioeconomic Inequalities in Heart Disease, Stroke, and Cardiovascular Disease Mortality Among Working Age Populations, United States, 1969-2011. *International Journal of MCH and AIDS*, *3*(2), 119–133. http://www.ncbi.nlm.nih.gov/pubmed/27621992

Slotman, A., Snijder, M. B., Ikram, U. Z., Schene, A. H., & Stevens, G. W. J. M. (2017). The role of mastery in the relationship between perceived ethnic discrimination and depression: The HELIUS study. *Cultural Diversity and Ethnic Minority Psychology*, *23*(2), 200–208. %22g.w.j.m.stevens@uu.nl%22]

Smith, D. M., Wang, S. B., Carter, M. L., Fox, K. R., & Hooley, J. M. (2020). Longitudinal predictors of self-injurious thoughts and behaviors in sexual and gender minority adolescents. *Journal of Abnormal Psychology*, *129*(1), 114–121. 0000-0002-8583-3014%22,

Smith, R., Drennan, V., Mackenzie, A., & Greenwood, N. (2018). The impact of befriending and peer support on family carers of people living with dementia: A mixed methods study. *Archives of Gerontology and Geriatrics*, *76*, 188–195. 0000-0002-2968-9169%22,

Sørensen, T., Kleiner, R., Ngo, P., Sørensen, A., & Bøe, N. (2013). From sociocultural disintegration to community connectedness dimensions of local community concepts and their effects on psychological health of its residents. *Psychiatry Journal*, *2013*, 1–13. https://doi.org/10.1155/2013/872146

Spahlholz, J., Pabst, A., Riedel-Heller, S. G., & Luck-Sikorski, C. (2016). Coping with perceived weight discrimination: Testing a theoretical model for examining the relationship between perceived weight discrimination and depressive symptoms in a representative sample of individuals with obesity. *International Journal of Obesity*, *40*(12), 1915–1921. %22Jenny.Spahlholz@medizin.uni-leipzig.de%22]

Stathopoulou, T., Avrami, L., Mouriki, A., Cavounidis, J., & Kostaki, A. (2018). Self-reported depression among migrant and native populations in Greece in times of crisis. *European Journal of Public Health*, *28*, 32–37. 0000-0002-5528-216X%22,

Steel, J. L., Dunlavy, A. C., Harding, C. E., & Theorell, T. (2017). The Psychological Consequences of Pre-Emigration Trauma and Post-Migration Stress in Refugees and Immigrants from Africa. *Journal of Immigrant and Minority Health*, *19*(3), 523–532. https://doi.org/10.1007/S10903-016-0478-Z

Stepanikova, I., & Kukla, L. (2017). Is perceived discrimination in pregnancy prospectively linked to postpartum depression? Exploring the role of education. *Maternal and Child Health Journal*, *21*(8), 1669–1677. %22irena@uab.edu%22]

Stojanovski, K., Zhou, S., King, E., Gjorgjiovska, J., & Mihajlov, A. (2018). An application of the minority stress model in a non-Western context: Discrimination and mental health among sexual and gender minorities in Macedonia. *Sexuality Research & Social Policy: A Journal of the NSRC*, *15*(3), 367–376. 0000-0003-1239-5153%22,

Stronks, K., Şekercan, A., Snijder, M., Lok, A., Verhoeff, A. P., Kunst, A. E., & Galenkamp, H. (2020). Higher prevalence of depressed mood in immigrants’ offspring reflects their social conditions in the host country: The HELIUS study. *PLoS ONE*, *15*(6). 0000-0002-0921-2232%22,

Swanson, V., Sharpe, T., Porteous, C., Hunter, C., & Shearer, D. (2016). Indoor annual sunlight opportunity in domestic dwellings may predict well-being in urban residents in Scotland. *Ecopsychology*, *8*(2), 121–130. 0000-0002-1685-2991%22,

Tan, S.-Y., & Haining, R. (2016). Crime victimization and the implications for individual health and wellbeing: A Sheffield case study. *Social Science & Medicine*, *167*, 128–139. %22bob.haining@geog.cam.ac.uk%22,

Taylor, L. K., Merrilees, C. E., Cairns, E., Shirlow, P., Goeke-Morey, M., & Cummings, E. M. (2013). Risk and resilience: The moderating role of social coping for maternal mental health in a setting of political conflict. *International Journal of Psychology*, *48*(4), 591–603. %22ltaylo12@nd.edu%22]

Thomas, F. (2015). The role of natural environments within women’s everyday health and wellbeing in Copenhagen, Denmark. *Health & Place*, *35*, 187–195. %22f.thomas@exeter.ac.uk%22]

Thoresen, S., Birkeland, M. S., Wentzel-Larsen, T., & Blix, I. (2018). Loss of trust may never heal Institutional trust in disaster victims in a long-term perspective: Associations with social support and mental health. *Frontiers in Psychology*, *9*. %22siri.thoresen@nkvts.no%22]

Timmins, L., Rimes, K. A., & Rahman, Q. (2018). Minority stressors, rumination, and psychological distress in monozygotic twins discordant for sexual minority status. *Psychological Medicine*, *48*(10), 1705–1712. 0000-0001-8346-4529%22,

van den Berg, M. M., van Poppel, M., van Kamp, I., Ruijsbroek, A., Triguero-Mas, M., Gidlow, C., Nieuwenhuijsen, M. J., Gražulevičiene, R., van Mechelen, W., Kruize, H., & Maas, J. (2019). Do physical activity, social cohesion, and loneliness mediate the association between time spent visiting green space and mental health? *Environment and Behavior*, *51*(2), 144–166. 0000-0002-0210-8053%22,

van den Berg, M., van Poppel, M., van Kamp, I., Andrusaityte, S., Balseviciene, B., Cirach, M., Danileviciute, A., Ellis, N., Hurst, G., Masterson, D., Smith, G., Triguero-Mas, M., Uzdanaviciute, I., de Wit, P., van Mechelen, W., Gidlow, C., Grazuleviciene, R., Nieuwenhuijsen, M. J., Kruize, H., & Maas, J. (2016). Visiting green space is associated with mental health and vitality: A cross-sectional study in four European cities. *Health & Place*, *38*, 8–15. %22mm.vandenberg@vumc.nl%22]

Verelst, A., Spaas, C., Pfeiffer, E., Devlieger, I., Kankaapää, R., Peltonen, K., Vänskä, M., Soye, E., Watters, C., Osman, F., Durbeej, N., Sarkadi, A., Andersen, A., Primdahl, N. L., & Derluyn, I. (2022). Social determinants of the mental health of young migrants. *European Journal of Health Psychology*, *29*(1), 61–73. 0000-0001-5450-1239%22,

Vilhjalmsdottir, A., Gardarsdottir, R. B., Bernburg, J. G., & Sigfusdottir, I. D. (2016). Neighborhood income inequality, social capital and emotional distress among adolescents: A population-based study. *Journal of Adolescence*, *51*, 92–102. 0000-0003-3368-4616%22,

Voci, A., Hadziosmanovic, E., Cakal, H., Veneziani, C. A., & Hewstone, M. (2017). Impact of pre-war and post-war intergroup contact on intergroup relations and mental health: Evidence from a Bosnian sample. *Peace and Conflict: Journal of Peace Psychology*, *23*(3), 250–259. 0000-0002-8861-0465%22,

Weimann, H., Rylander, L., Albin, M., Skärbäck, E., Grahn, P., Östergren, P.-O., & Björk, J. (2015). Effects of changing exposure to neighbourhood greenness on general and mental health: A longitudinal study. *Health & Place*, *33*, 48–56. %22jonas.bjork@med.lu.se%22,

White, M. P., Alcock, I., Wheeler, B. W., & Depledge, M. H. (2013). Would you be happier living in a greener urban area? A fixed-effects analysis of panel data. *Psychological Science*, *24*(6), 920–928. 0000-0001-9404-5936%22,

Wickham, S., Shryane, N., Lyons, M., Dickins, T., & Bentall, R. (2014). Why does relative deprivation affect mental health? The role of justice, trust and social rank in psychological wellbeing and paranoid ideation. *Journal of Public Mental Health*, *13*(2), 114–126. %22slw@liverpool.ac.uk%22]

Wiginton, J. M., Murray, S., Kall, M., Maksut, J. L., Augustinavicius, J., Delpech, V., & Baral, S. D. (2021). HIV-related stigma and discrimination in health care and health-related quality of life among people living with HIV in England and Wales: A latent class analysis. *Stigma and Health*. 0000-0001-6971-427X%22,

Wind, T. R., & Komproe, I. H. (2012). The mechanisms that associate community social capital with post-disaster mental health: A multilevel model. *Social Science & Medicine*, *75*(9), 1715–1720. %22twind@healthnettpo.org%22]

Wothge, J., & Niemann, H. (2020). Gesundheitliche Auswirkungen von Umgebungslärm im urbanen Raum Einleitung. *Bundesgesundheitsblatt - Gesundheitsforschung - Gesundheitsschutz*, *63*, 987–996. https://doi.org/10.1007/s00103-020-03178-9

Wu, Y.-T., Prina, A. M., Jones, A., Barnes, L. E., Matthews, F. E., & Brayne, C. (2017). Micro-scale environment and mental health in later life: Results from the Cognitive Function and Ageing Study II (CFAS II). *Journal of Affective Disorders*, *218*, 359–364. %22y.wu3@exeter.ac.uk%22]

Wypych, M., & Bilewicz, M. (2022). Psychological toll of hate speech: The role of acculturation stress in the effects of exposure to ethnic slurs on mental health among Ukrainian immigrants in Poland. *Cultural Diversity and Ethnic Minority Psychology*. 0000-0001-5027-1691%22,

Ye, J., Wen, Y., Sun, X., Chu, X., Li, P., Cheng, B., Cheng, S., Liu, L., Zhang, L., Ma, M., Qi, X., Liang, C., Kafle, O. P., Jia, Y., Wu, C., Wang, S., Wang, X., Ning, Y., Sun, S., & Zhang, F. (2021). Socioeconomic deprivation index is associated with psychiatric disorders: An observational and genome-wide gene-by-environment interaction analysis in the UK Biobank cohort. *Biological Psychiatry*, *89*(9), 888–895. 0000-0003-1339-5956%22,

Zhou, S., King, E. J., Gjorgiovska, J., Mihajlov, A., & Stojanovski, K. (2019). Self-concealment, discrimination, and mental health in Macedonia: Disparities experienced by sexual and gender minorities. *Global Public Health: An International Journal for Research, Policy and Practice*, *14*(8), 1075–1086. 0000-0003-1239-5153%22,
